# A 4-methyl-substituted durlobactam analogue as a potential class-D oxacillinase inhibitor in *Acinetobacter baumannii—*an *in silico* study

**DOI:** 10.3389/fbinf.2026.1790411

**Published:** 2026-05-26

**Authors:** Elizabeth Annie George, Aniket Naha, Sudha Ramaiah

**Affiliations:** 1 Medical and Biological Computing Laboratory, School of Bio-Sciences and Technology (SBST), Vellore Institute of Technology, Vellore, India; 2 Department of Bio-Sciences, School of Bio-Sciences and Technology, Vellore Institute of Technology (VIT), Vellore, Tamil Nadu, India; 3 Medical Biotechnology and Computational Drug Designing Laboratory, Pushpagiri Institute of Medical Sciences and Research Centre, Pushpagiri Medical Society, Tiruvalla, Kerala, India

**Keywords:** *Acinetobacter baumannii*, molecular dynamics simulation, oxacillinases, quantum chemical simulations, S-X-X-K motif

## Abstract

The emergence of extensively drug-resistant *Acinetobacter baumannii* expressing oxacillinases poses a significant challenge in nosocomial settings. Despite durlobactam being a potent β-lactam inhibitor, its long-term efficacy is limited, particularly against class-D β-lactamase-producing strains. In this study, an integrated computational approach was utilized to characterize a library of durlobactam analogues against two expressed class-D oxacillinases (OXA23 and OXA58) from the whole-genome sequences of pan-Indian clinical isolates. Tanimoto coefficient-based virtual screening identified analogues retaining the key pharmacophoric features of durlobactam. Pharmacokinetics and toxicity profiling indicated favourable drug-like properties with minimal predicted toxicity. Molecular docking and interaction analyses revealed a 4-methyl-substituted durlobactam analogue (CHEMBL3140306) exhibiting strong interactions with active serine residues (S79 for OXA23 and S83 for OXA58) within the conserved S-X-X-K motif that is critical for enzymatic activity of both β-lactamases. Quantum chemical simulations supported the structural stability and favourable reactivity profile of the predicted lead molecule. Molecular dynamics simulation further demonstrated stable and compact binding, characterized by persistent hydrogen bonding and a favourable thermodynamics profile. Binding free calculations and essential dynamics corroborated the affinity and conformational flexibility across both oxacillinases. Overall, in this computational study, we identify a 4-methyl-substituted durlobactam analogue as a promising exploratory inhibitor of oxacillinases, thus offering a basis for further experimental validations.

## 1 Introduction

*Acinetobacter baumannii*, an opportunistic, Gram-negative, non-fermentative coccobacillus, has been identified as a significant cause of nosocomial outbreaks, including urinary tract infections, ventilator-associated pneumonia, and bloodstream and wound infections (Sindhu, 2018; Naha et al., 2021). It ranks among the top five pathogens associated with antimicrobial resistance (AMR)-related fatalities and has been ranked as a priority-1 critical pathogen by the World Health Organization (WHO) (Murray et al., 2022; Karah et al., 2023). It causes opportunistic infections, especially in the immunocompromised patients within intensive care units (ICUs), by exploiting breaches in the body’s anatomical barriers (Anitha et al., 2014; Bassetti and Giacobbe, 2020). The production of β-lactamases, especially class-D carbapenem hydrolysing oxacillinases such as OXA23 and OXA58, effectively hydrolyses the β-lactam (βL) ring of the antibiotics, rendering them ineffective (Pearl and Anbarasu, 2025). The effectiveness of the last-resort antibiotics such as carbapenems has significantly decreased because of this enzymatic degradation (El-Ghali et al., 2023; Panickar et al., 2024). Consequently, β-lactamase inhibitors (βLIs) have drawn significant attention as a potential strategy to restore the βL activity by blocking these enzymes (Vijayakumar et al., 2024; Zhang et al., 2024).

Among these, durlobactam, previously referred to as ETX2514, is a novel diazabicyclooctane (DBO) βLI that exhibits potent activity against serine β-lactamases, including the OXA-type carbapenemases (Papp-Wallace et al., 2023). Durlobactam is currently undergoing clinical evaluation and shows promising synergy with sulbactam, a βL antibiotic possessing intrinsic resistance to this pathogen (Spernovasilis et al., 2025). This combined βL–βLI antibiotic has been approved by the United States Food and Drug Administration (FDA) for the treatment of severe infections, including hospital-acquired and ventilator-associated bacterial pneumonia caused by multidrug-resistant (MDR) and extensively drug-resistant (XDR) members of *A. baumannii* calcoaceticus (*ABC*) complex (Kaye et al., 2023). Although the *ABC* complex consists of several closely related species, namely, *A. baumannii*, *Acinetobacter nosocomialis*, and *Acinetobacter pitti*, *A. baumannii* is the most clinically dominant member associated with carbapenem-mediated resistance by OXA-type β-lactamases. Although sulbactam–durlobactam has been reported as a potential combination therapy against MDR *A. baumannii,* recent studies reported that ∼2.3% of the strains have started demonstrating resistance profiles to this combination (Principe et al., 2022; Segatore et al., 2022). Similarly, 2% of 5,032 clinical isolates of *ABC* collected from 2016 to 2021 in a global surveillance study showed the minimum inhibitory concentration (MIC) values > 4 μg/mL this combination, indicating the trend of decreasing susceptibility in the upcoming years (Moussa et al., 2023; O’Donnell et al., 2024).

Despite its therapeutic potential, the emergence of resistance towards this antibiotic is still a growing concern, and this highlights the urgent need to develop structural analogues with improved efficacy and stability against class-D β-lactamases (Malathi et al., 2017; Principe et al., 2022). The structural modification of durlobactam through small alkyl substitutions, especially 4-methyl substitution, represents a promising strategy to target the tunnel-like hydrophobic cleft at the active-site entrance formed by flexible loops and the non-polar residues (Shapiro et al., 2021). These substitutions are expected to enhance the pi–alkyl and hydrophobic interactions with aromatic side chains, thus improving the acyl–enzymes complex stabilization while sterically restricting the hydrolytic water channels involved in deacylation-mediated resistance (Papp-Wallace et al., 2023). In the current study, we aimed to develop and assess novel analogues that specifically target OXA23 and OXA58, which are present in nosocomial pan-India isolates. A comprehensive *in silico* approach integrating cheminformatics and structural bioinformatics approaches was utilized to identify an exploratory βLI. This computational framework enabled the systematic evaluation of molecular characteristics, binding properties, and the dynamic stability of the lead molecules. In this study, we seek to explore a promising βLI that could serve as a therapeutic alternative against MDR *A. baumannii* upon further experimental validation.

## 2 Materials and methods

### 2.1 Identification of the druggable targets of *Acinetobacter baumannii,* and the stability assessments

Whole-genome sequencing (WGS) from 28 non-duplicate *A. baumannii* nosocomial strains generated from our previous study was analysed (Naha et al., 2021). These isolates were obtained from the blood (*n* = 21) and sputum (*n* = 7), which were collected at Christian Medical College, Vellore. All the strains were previously identified and characterized as XDR based on the phenotypic antibiotic susceptibility testing and molecular characterization, as described in our previous study involving WGS and the evaluation of a novel sulbactam–durlobactam combination. From the dataset, class-D serine β-lactamase targets, specifically OXA23 and OXA58, were selected as potential druggable targets. The OXA23 and OXA58 derived from the WGS data were modelled using the suitable PDB templates 5WI3 and 4OH0, respectively, and optimized in the previous study (Naha et al., 2021). These optimized 3D structures of OXA23 and OXA58 were considered for the current study.

To assess the stability of the protein, molecular dynamics simulations were performed, and topology was built using *GROMACS 2024.2* package CHARMM36-Jul2022 force-field (Naha et al., 2022; George et al., 2025a). The targets were solvated in TIP3P water model within cubic boxes (1.0 nm), neutralized with counterions (Na^+^/Cl^−^), energy minimized (50,000 steps), and equilibrated under standard NVT and NPT conditions (100 ps each) (Naha and Ramaiah, 2023). A 100,000-ps (100 ns) MD run was performed, and the stability parameters were assessed.

### 2.2 Virtual screening of durlobactam analogues

Based on text mining and drug bank screening, the conventional βLI durlobactam (PubChem CID: 89851852) was considered as the reference molecule. Rapid ligand-based virtual screening was performed for ChEMBL-approved drugs and commercial (ZINC) and bioactive compounds libraries utilizing SwissSimilarity webserver (Bragina et al., 2022). The similarity was quantified by the ratio called the Tanimoto coefficient, and the molecular fingerprints were generated based on structural homology descriptors (Naha et al., 2022). This similarity-based filtering approach computed by the Tanimoto coefficient was utilized to shortlist the leads.

### 2.3 Pharmacokinetic filtering of analogues

The physicochemical descriptors such as the pharmacokinetics parameters such as absorption, distribution, metabolism, excretion (ADME), drug-likeness, and medicinal chemistry of the lead molecule were predicted using SwissADME server, and the results were compared with the reference compound (Daina et al., 2017). The *in silico* toxicity predictions including the LD_50_, toxicity class, and toxicity endpoints were assessed from the ProTox 3.0 server using pharmacophore modelling, molecular homology, and machine learning algorithms (Banerjee et al., 2024).

### 2.4 Molecular docking analysis and intermolecular Interaction profiling

The best ligands were identified through a turnkey computational docking program, AutoDock Vina 1.2.0, which utilizes a simple scoring function and rapid gradient-optimization search to efficiently predict binding affinities (Eberhardt et al., 2021). The best-performing ligands were further subjected to docking simulations to refine and confirm the interactions with the targets, namely, OXA23 and OXA58 (Naha et al., 2021). This iterative approach ensured the selection of the most promising lead molecule. Site-specific molecular docking was performed targeting the active site residues, as described by Naha et al., using AutoDock 4.2 (Naha et al., 2021). The drug target was optimized by the addition of polar hydrogen atoms and subsequently merging non-polar atoms followed by the addition of required Kollman charges. Similarly, ligand torsions were fixed prior to docking by merging non-polar hydrogens and assigning Gasteiger charges. The active site of the protein was centred within an affinity grid box of uniform dimension (60 × 60 × 60 Å^3^), with 0.375 Å grid point spacing. The Autogrid4 and Autodock4 programs were utilized to generate protein–ligand complexes. Finally, Lamarckian and genetic algorithms were used for choosing the complexes with the least binding energy (Morris et al., 2008; Naha et al., 2022). Molecular dockings were conducted in triplicates for the classical βLI, and each of the shortlisted leads and the results were expressed as the mean ± standard deviation (SD) to ensure reliability. The intermolecular interaction profiles of the docked complexes were visualized in Discovery Studio 2024 v24.1.0.23298 (Baroroh et al., 2023).

### 2.5 Quantum chemical calculations and ligand optimization

The chemical reactivity, intrinsic properties, and the stability of the leads that demonstrated the least binding affinities upon binding with their drug targets were meticulously ascertained through advanced density function theory (DFT) calculations (Usharani et al., 2023; Nagarajan et al., 2025). The Gaussian-09 computational chemistry standalone software package was used for ligand structure optimization, and the results were subsequently visualized in GaussView v6.0.16 software (Frisch et al., 2009; Dennington et al., 2016). Molecular geometry optimization was performed to achieve stable conformations with minimal energy using Becke’s three-parameter (B3) exchange-correlation function conjugated with the Lee–Yang–Parr (LYP) method and the 6-311G ++ (d,p) basis set (Kohn and Sham, 1965; Stephens et al., 1994). The current basis state was found to be ideal for the analysis as polarization was achieved with p-type functional being added to all hydrogen atoms, while d-type diffused functional was added to the electronegative atoms possessing lone pairs such as nitrogen, oxygen, and sulphur. The quantum chemical simulations were carried out in Dell Precision Tower 3660 Workstation equipped with 12th generation Intel Core i9-12900 (2.40 GHz) processor and 88 GB RAM layout.

The lowest energy conformer attainment and density-of-states (DOS) graphs were calculated using the GaussSum v3.0, which provides insights into electron density distribution across the different energy levels (O’boyle et al., 2008; Fei et al., 2021). The intrinsic behaviour and the chemical reactivity of the leads were determined using frontier molecular orbitals (FMO), natural bond orbitals (NBO), and Laplacian electrostatic potential (ESP) maps (Usharani et al., 2023; Nagarajan et al., 2025). The FMO analysis represented the electronic shifts that occur from the highest occupied molecular orbital (HOMO) to the lowest unoccupied molecular orbital (LUMO). The energy gap (ΔE), ionization energy (I), and electron affinity (A) were calculated to understand the molecules’ redox potential, stability, and reactivity (Joshi et al., 2014). Additional insights into charge delocalization and electron density transfer were obtained by assessing the presence of electropositive and electronegative centres within the molecule using ESP. The ESP was mapped over the total density using the B3LYP/6-311G++(d,p) level of theory. The isosurfaces were visualised in the GaussView, while the contour and projection maps were generated using Multiwfn software. The NBO analysis determines the strength of intermolecular interactions and the extent of delocalized electron density by measuring the charge delocalization from bonding (electronegative donor) to antibonding* (electron-deficient acceptor) atomic orbitals (i, j). The stability of these interactions is expressed in terms of the stabilization energy (E^(2)^), which is calculated using the Fock matrix derived from second-order perturbation theory (Naha et al., 2022). Additionally, various quantum theoretical parameters, including electronegativity (χ), chemical potential (ρ), chemical hardness (η), chemical softness (δ), global electrophilicity index (ω), and global nucleophilicity index (N) were calculated in accordance to Koopmans’ Theorem (Usharani et al., 2023; Nagarajan et al., 2025).

The multifunctional wavefunction analysis software Multiwfn v3.8 was used for the wavefunction and topology analyses, encompassing the electron localization function (ELF) and localised orbital locator (LOL), to identify the reactive centres of the leads (Lu and Chen, 2012). The pharmacodynamics parameters of the leads were analysed through the lenses of the static thermodynamic functions such as enthalpy (H), entropy (S), heat capacity (Cp), and Gibbs free energy (G) under the effect of increasing temperature (25 K–1,000 K). The pharmacodynamics calculations were inferred under the hybrid functionals B3LYP with the numerical polarization (DNP) basic set using the DMOL3 module in BIOVIA Materials Studio 2020 v20.1.0.2728 (Meunier and Robertson, 2021).

### 2.6 Evaluation of stability of protein-ligand complexes

The stability of the best protein–ligand complexes was validated via all-atom molecular dynamics simulation (MDS) using the GROningen MAchine for Chemical Simulation (GROMACS) suite (Lemkul, 2019). It is a flexible open-source MDS suite optimized for protein-in-water to complex protein–ligand simulations (Lemkul, 2024). The protein–ligand simulations were carried out for a period of 100,000 ps (100 ns) by simulating an aqueous cellular environment utilizing the GROMACS 2024.2 package (George et al., 2025b). The topology of the protein was build using CHARMM36 (July 2022) force-field mechanics. It was solvated by centring the protein within a dodecahedron box with 1.0-nm uniform dimension, and the complex was neutralized by the addition of Na^+^ or Cl^−^ counterions in 50,000 steps. Energy minimization was performed using the steepest descent algorithm and F_max_ < 1,000 kJ/mol nm^-1^ (Naha et al., 2022). Furthermore, the system underwent two-phase equilibration for 100 ps under a constant number of particles, volume, and temperature (NVT) and the constant number of particles, pressure, and temperature (NPT) ensembles. The temperature of the system was maintained at 300 K using a thermostat, and pressure was monitored using the barostat, allowing density stabilization. The system equilibration was assessed by monitoring the thermodynamic stability parameters (temperature, pressure, and density) to ensure adequate relaxation of the system (Mohapatra et al., 2023). This equilibrated system was then subjected to an MD run for 100,000 ps using particle–particle (PP) and particle–mesh Ewald (PME) calculations for three independent MD runs (George et al., 2025a). The MD trajectories thus obtained were analysed and reported in terms of the mean ± SD and visualized using the Grace software to evaluate the structural stability and dynamic behaviour of the complexes (Jayaraman et al., 2019; Nagarajan et al., 2025).

### 2.7 Ligand-binding assessment using protein ensemble docking

To explore the dynamic interactions of the ligands with time-dependent conformation targets, protein structures were removed from the 100-ns protein simulation trajectories at every 10 ns (0 ns–100 ns), thus extracting 11 different protein conformations or frames to capture the structural variability during the simulation timeframe. The structures were pre-processed, thus optimizing their geometries by adding polar hydrogens and Kollman charges using AutoDock 4.2 (Morris et al., 2008). Each frame was independently docked with the ligands targeting their active sites. The binding affinities of the ligands were assessed, and changes in the interactions were evaluated as a function of conformational dynamics.

### 2.8 Principal component analysis (PCA)-based free energy landscape (FEL) profiling

To characterize the high-amplitude collective motions of the protein–ligand complexes, essential dynamics analysis was carried out over a timeframe of 100 ns in the GROMACS 2024.2 package (Jayaraman et al., 2019). To minimize the statistical noise and emphasize the backbone dynamics, covariance matrix was generated from Cα atoms. The magnitude of PCA was represented in terms of its eigenvectors and their corresponding eigenvalues. The cosine content of the principal components ranging from 0 to 1 (no cosine–perfect cosine) was further used to evaluate the significance of these motions (George et al., 2025b). The first two components were analysed in order to interpret the conformational space and identify stable and low-energy cluster basins. The covariance matrix was constructed using the *gmx_covar* module, eigenvectors were plotted using *gmx_anaeig*, and 2D and 3D maps of the protein were plotted against the first two principal components (PC1 and PC2) utilizing the *gmx_sham* script (Mittal et al., 2021; Kumari et al., 2022). The PCA-based FEL depicts the thermodynamic conformational profile of the system in terms of Gibbs free energy derived from the extensive collective motions that occurred during the MD simulations, where the low-energy basins correspond to stable and highly populated conformations, while the higher energy corresponds to less favourable translational conformations (Papaleo et al., 2009).

### 2.9 Binding free energy estimation of protein–ligand complexes

The binding free energy calculations of the protein–ligand complexes were evaluated using molecular mechanics-generalized Born surface area (MM-GBSA) analysis using *gmx_MMPBSA* within the GROMACS suite (Valdés-Tresanco et al., 2021). This approach integrates molecular mechanic energies with a solvation model based on generalized Born approximation and surface area. All the simulations were performed at the temperature 298.15 K (25 °C) using the solvation parameter (igb) of 5 and, the internal and external dielectric constants were defined as 1.0 and 78.5, respectively (George et al., 2025a). The total binding free energy (ΔG_*Binding*_) was estimated over the 100-ns MDS trajectory based to the following equation:

$${\Delta G}_{Binding}={\Delta G}_{Complex}-(+{\Delta G}_{Ligand}),$$

where ΔG_*Complex*_, ΔG_*Protein*_, and ΔG_*Ligand*_ correspond to the free energies of the bound complex, receptor protein, and ligand, respectively.

## 3 Results

### 3.1 Selection of the druggable targets and stability assessments

**FIGURE 1:**
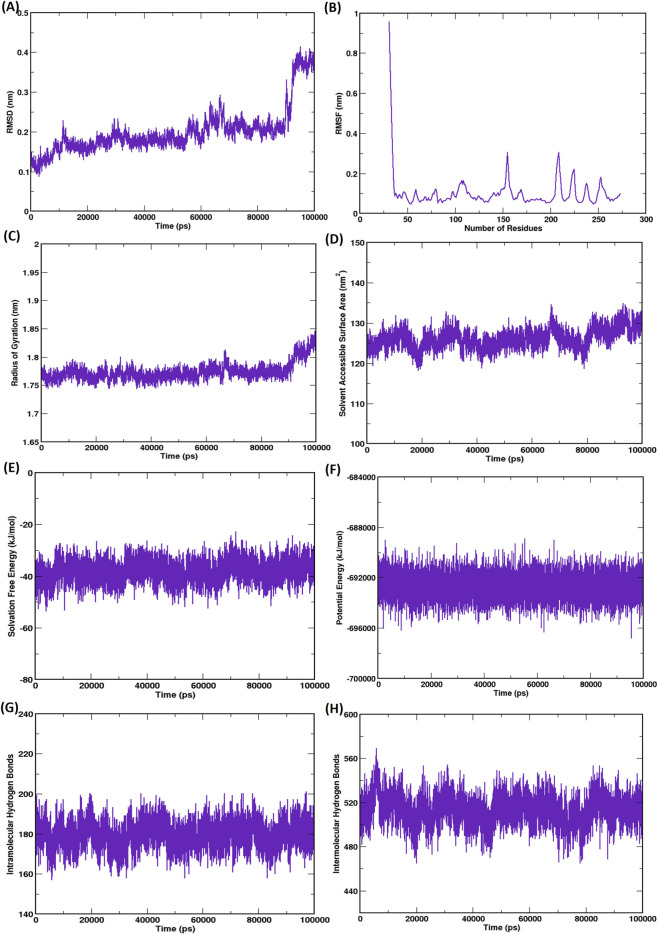
All-atom molecular dynamics simulation (MDS) of OXA23 **(A)** RMSD; **(B)** RMSF; **(C)** Rg plot; **(D)** SASA; **(E)** solvation free energy; **(F)** potential energy; **(G)** intramolecular hydrogen bond; **(H)** intermolecular hydrogen bond.

**FIGURE 2:**
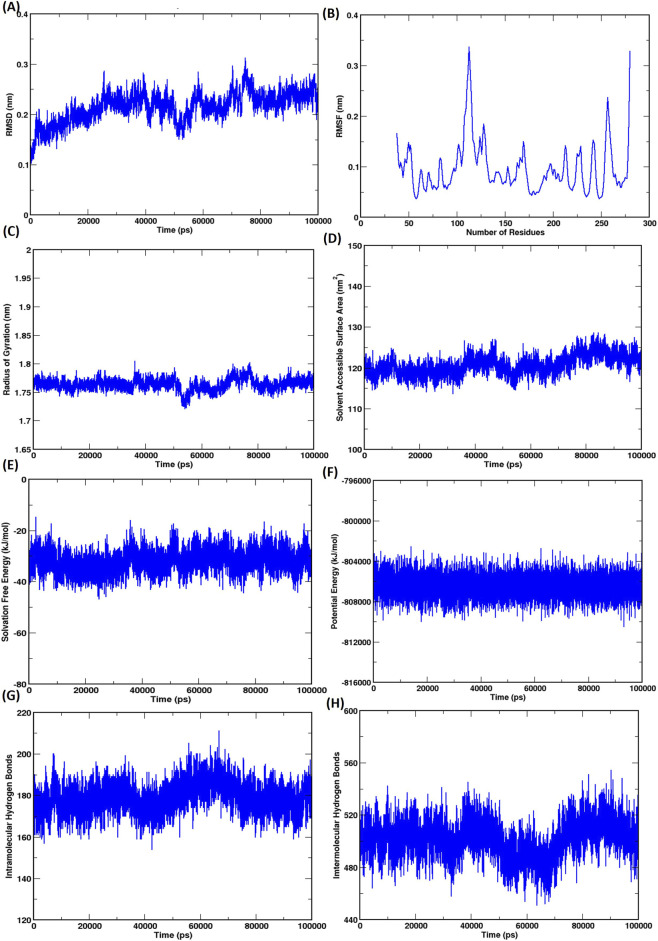
All-atom molecular dynamics simulation (MDS) of OXA58 **(A)** RMSD; **(B)** RMSF; **(C)** Rg plot; **(D)** SASA; **(E)** solvation free energy; **(F)** potential energy; **(G)** intramolecular hydrogen bond; **(H)** intermolecular hydrogen bond.

All the 28 isolates demonstrated resistance to sulbactam, with a minimum inhibitory concentration (MIC) value between 8 and 256 μg/mL. Both the MIC_50_ and MIC_90_ values were 256 μg/mL, indicating high resistance across the strains. Analysis of oxacillinases landscape revealed the expression of the *bla*_*OXA23*_ and *bla*_*OXA58*_ genes. The optimized 3D structures of OXA23 and OXA58 targets procured from our previous study were further subjected to stability assessments using various MDS metrices. It was observed that OXA23 displayed a lower root mean square deviation (RMSD) of 0.20 ± 0.06 nm (Figure 1A) and root mean square fluctuations (RMSFs) of 0.10 ± 0.09 nm, revealing the limited backbone and residue-level flexibility (Figure 1B). In addition to this, it maintained a compact structure with a radius of gyration (Rg) of 1.77 ± 0.01 nm (Figure 1C), displayed solvent-accessible surface area (SASA) of 126 ± 2.44 nm^2^ (Figure 1D), showed a favourable solvation free energy of −38.21 ± 4.01 kcal/mol (Figure 1E), and exhibited a potential energy of −692,696.92 kcal/mol (Figure 1F), which was further supported by the formation of 179 intramolecular (Figure 1G) and 513 intermolecular hydrogen bonds confirming its confirmational stability (Figure 1H). Similarly, OXA58 also demonstrated comparable stability with RMSD of 0.21 ± 0.03 nm (Figure 2A) and RMSF of 0.10 ± 0.05 nm (Figure 2B), along with Rg of 1.76 ± 0.01 nm (Figure 2C). It exhibited lower SASA of 120 ± 2.37 nm^2^ (Figure 2D) and solvation free energy of −31.89 ± 4.12 kcal/mol (Figure 2E). It displayed a favourable potential energy of −692,696.92 kcal/mol (Figure 2F) and 179 intramolecular (Figure 2G) and 501 intermolecular hydrogen bonds (Figure 2H), thus reflecting the stability of the protein targets.

### 3.2 Retrieval of durlobactam analogues

Ligand-based virtual screening approach was utilized to identify the structural analogues of the reference compound, durlobactam, leading to a library comprising of 1,217 analogues (Supplementary Material). To prioritize them, the compounds for the analogues were initially ranked based on the fingerprint-based structural similarity metric Tanimoto coefficient. The top-15 unique leads that exhibited a Tanimoto coefficient greater than 0.97 were shortlisted, ensuring that the analogues were likely to retain key pharmacophoric features while allowing minute side-chain variations of the parent compound.

### 3.3 Evaluation of pharmacokinetic ADME and toxicity profiles

The 15 unique shortlisted leads exhibited favourable physiochemical properties, including molecular weight, hydrogen bond donors and acceptors, topological surface area (TPSA), lipophilicity (LogP), and water solubility. The pharmacokinetic properties such as high LD_50_ values, with the minimal predicted toxicity risks, including hepatotoxicity, neurotoxicity, mutagenicity, and high gastrointestinal (GI) absorption; metabolic stability as the major cytochrome inhibitor; skin permeation; and bioavailability of majority of the leads aligns well with the reference compound. Moreover, most of the leads revealed no significant violations of the drug-likeness rules, especially Lipinski’s rule of five and Veber’s rules violations (Table 1). The detailed list of all the parameters of the shortlisted leads is furnished in Supplementary Material SI1.

**TABLE 1:** Pharmacokinetics profiling and drug-likeness evaluation of durlobactam and its analogues.

Properties	Dur	A1	A2	A3	A4	A5	A6	A7	A8	A9	A10	A11	A12	A13	A14	A15
Molecular weight	277.3	265.2	265.2	265.2	277.3	277.3	277.3	277.3	220.2	259.2	259.2	265.2	265.2	265.2	265.2	277.3
Molar refractivity	64.61	60.27	60.27	60.27	64.61	64.61	64.61	64.61	52.09	61.45	61.45	60.27	60.27	60.27	60.27	62.97
TPSA	138.6	138.6	138.6	138.6	138.6	138.6	138.6	138.6	95.5	119.3	119.3	138.6	138.6	138.6	138.6	138.6
iLOGP	0.64	0.16	−0.36	0.34	0.64	0.15	−0.24	0.26	0.45	0.64	1.24	0.16	−0.18	−0.36	0.34	−0.25
GI absorption	Low	Low	Low	Low	Low	Low	Low	Low	High	High	High	Low	Low	Low	Low	Low
PGP substrate	No	No	No	No	No	No	No	No	No	No	No	No	No	No	No	Yes
Lipinski violations	0	0	0	0	0	0	0	0	0	0	0	0	0	0	0	0
Veber violations	0	0	0	0	0	0	0	0	0	0	0	0	0	0	0	0
Bioavailability score	0.56	0.56	0.56	0.56	0.56	0.56	0.56	0.56	0.56	0.56	0.56	0.56	0.56	0.56	0.56	0.56
PAINS alerts	0	0	0	0	0	0	0	0	0	0	0	0	0	0	0	0
Brenk alerts	2	1	1	1	2	2	2	2	2	2	2	1	1	1	1	1
Lead-likeness violations	0	0	0	0	0	0	0	0	1	0	0	0	0	0	0	0
Synthetic accessibility	5.04	4.43	4.43	4.43	5.04	5.04	5.04	5.04	4.58	4.92	4.92	4.43	4.43	4.43	4.43	4.55
LD_50_ (mg/kg)	1500	1500	1500	1500	1500	1500	1500	1500	710	1500	1500	1500	1500	1500	1500	1500
Toxicity class	4	4	4	4	4	4	4	4	4	4	4	4	4	4	4	4
Hepatotoxicity	In	In	In	In	In	In	In	In	In	In	In	In	In	In	In	In
Neurotoxicity	In	In	In	In	In	In	In	In	In	In	In	In	In	In	In	In
Nephrotoxicity	In	In	In	In	In	In	In	In	In	Act	Act	In	In	In	In	In
Cardiotoxicity	In	In	In	In	In	In	In	In	In	In	In	In	In	In	In	In
Carcinogenicity	In	In	In	In	In	In	In	In	In	In	In	In	In	In	In	In
Immunotoxicity	In	In	In	In	In	In	In	In	In	In	In	In	In	In	In	In
Mutagenicity	In	In	In	In	In	In	In	In	In	In	In	In	In	In	In	In
Cytotoxicity	In	In	In	In	In	In	In	In	In	In	In	In	In	In	In	In
BBB-barrier	In	Act	Act	Act	Act	In	In	In	Act	In	In	Act	Act	Act	Act	Act
Interpretation	Safe	Safe	Safe	Safe	Safe	Safe	Safe	Safe	Safe	Safe	Safe	Safe	Safe	Safe	Safe	Safe

A, analogue; TPSA, topological polar surface area; GI, gastrointestinal; PGP, P-glycoprotein; LD, lethal dose; BBB, blood–brain barrier; IN, inactive; ACT, active.

### 3.4 Evaluation of the binding affinity of the analogues

**FIGURE 3:**
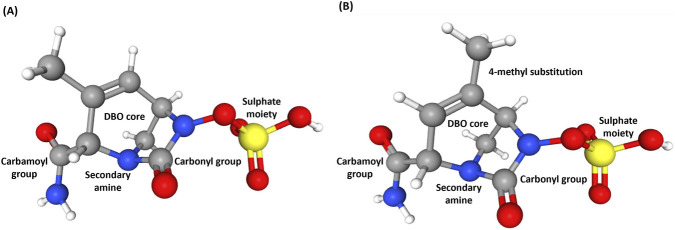
Structural representation of **(A)** durlobactam; **(B)** A7 highlighting the diazabicyclooctane (DBO) core, carbamoyl (-CONH_2_) group, secondary amine, carbonyl (-C=O) group, sulphate (-OSO_3_H) moiety, and 4-methyl-substitution of at the C4 position of A7.

From the initial set of 16 leads, the top-five analogues for each target protein were shortlisted based on their binding affinity scores, which indicates the strength of the protein–ligand interaction as computed using AutoDock Vina (Supplementary Material SI2). These selected ligands were subsequently subjected to site-specific molecular docking to assess their binding potential within the active sites of key class-D β-lactamase targets (OXA23 and OXA58). Molecular docking analysis revealed that the 4-methyl-substituted durlobactam analogue A7 (CHEMBL3140306) consistently exhibited superior binding across the tested targets when compared to the other analogues and the reference compound, durlobactam. Specifically, A7 exhibited a binding energy of −7.03 ± 0.16 kcal/mol against OXA23, indicating stronger interaction than that with durlobactam (−6.21 ± 0.33), followed by A16 (−6.53 ± 0.04 kcal/mol) and A8 (−6.15 ± 0.12 kcal/mol). Similarly in the case of OXA58, A7 was better than durlobactam (−6.52 ± 0.17 kcal/mol), demonstrating superior binding affinity with the binding energy of −7.33 ± 0.03 kcal/mol, which was followed by A6 (−7.31 ± 0.03 kcal/mol), A8 (−7.29 ± 0.13 kcal/mol), and A16 (−6.71 ± 0.09 kcal/mol), where the mean binding energies represent the averages from docking triplicates. The predicted binding affinities and the percentage of increment in the binding affinities of the docked complexes are tabulated in Table 2. Representation of the chemical structure of durlobactam and its 4-methyl-substituted analogue A7 are illustrated in Figure 3.

**TABLE 2:** Predicted binding affinities and inhibition constants for protein–ligand complexes.

Complex		Binding energy (kcal/mol)	Inhibition constant (uM)	Increment in binding affinity (%)
OXA23	Durlobactam	−6.21 ± 0.33	31.56	--
	A7	−7.03 ± 0.16	8.35	**13.20**
	A16	−6.53 ± 0.04	16.32	5.15
	A8	−6.15 ± 0.12	31.54	−0.97
	A13	−5.89 ± 0.25	51.20	−5.15
	A6	−5.80 ± 0.12	56.44	−6.60
OXA58	Durlobactam	−6.52 ± 0.17	19.98	--
	A7	−7.33 ± 0.03	9.65	**12.42**
	A6	−7.31 ± 0.03	4.4	12.12
	A8	−7.29 ± 0.13	4.55	11.81
	A16	−6.71 ± 0.09	12.12	2.91
	A13	−5.89 ± 0.17	49.80	−9.66

Binding energies are represented as the mean ± SD and are calculated from triplicate dockings. Percentage increment was calculated relative to the durlobactam based on the magnitude of binding energy. Negative increment in binding affinity indicates reduced binding affinity. Bold values indicate the compounds showing the highest increment in binding affinity compared with durlobactam.

### 3.5 Intermolecular interaction profiling of the complexes

**FIGURE 4:**
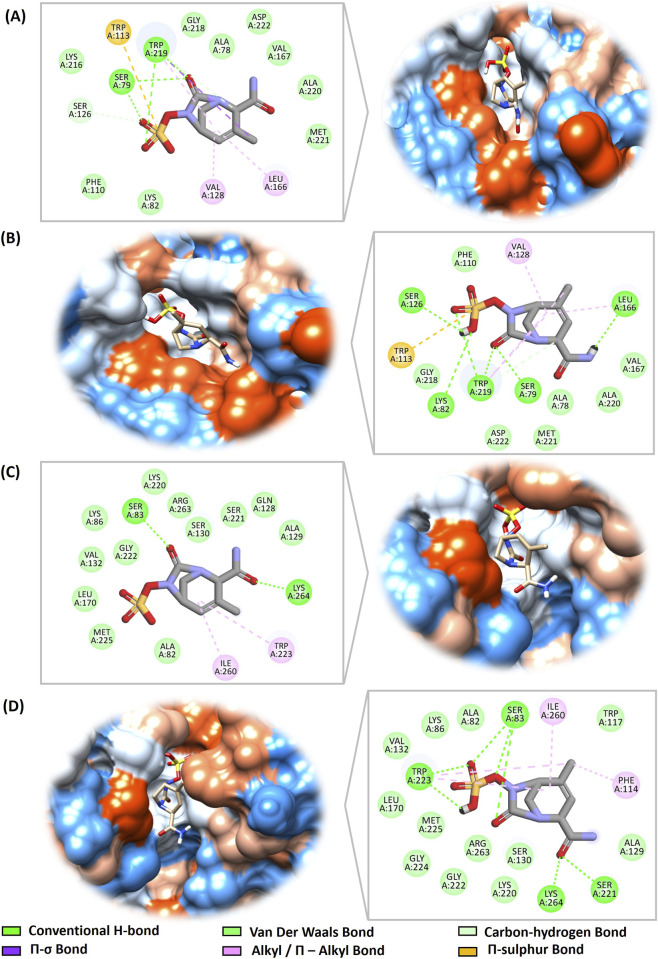
3D docked posed and 2D intermolecular interaction profile: **(A)** OXA23–durlobactam; **(B)** OXA23–A7; **(C)** OXA58–durlobactam; **(D)** OXA58–A7.

The intermolecular interactions between OXA23 residues and the reactive atoms of the durlobactam molecule revealed that durlobactam formed four conventional hydrogen bonds, involving interacting pairs of the residues S79–O3, W219–O3, S79–O6, and W219–O7. Additionally, two carbon–hydrogen bonds were observed with S126–O6 and W219–C13. The van der Waals interactions were noted with residues A220, A78, D222, F110, G218, K216, K82, M221, and V167. Moreover, durlobactam established π–sulphur interaction with W113, alkyl/π–alkyl interactions with L166, V128, and W219, and a π–σ interaction with W219 (Figure 4A). In the OXA23–A7 complex, A7 exhibited six conventional hydrogen bonds engaging the residues L166–N10, K82–O5, S126–O5, W219–O2, S79–O7, and W219–O7. It also formed a carbon–hydrogen bond with C13–W219. Additionally, it showed van der Waals interactions with A229, A78, D222, F110, G218, M221, and V167 and established a π–sulphur and alkyl/π–alkyl interactions with W113 and V128, respectively (Figure 4B).

For the OXA58–durlobactam complex, durlobactam formed conventional hydrogen bonds with residues S83–O3 and K264–O4. Additionally, it displayed van der Waals interactions with multiple residues, including A129, A82, G222, K220, K86, L170, M225, N128, R263, S130, S221, and V132. Alkyl/π–alkyl interactions were observed with I260 and W223 (Figure 4C). In OXA58–A7, A7 established seven conventional hydrogen bonds with residues S221–O4, K264–O4, S83–O3, S83–O2, S83–O6, W223–O6, and W223–O5. Furthermore, van der Waals interactions were recorded with residues A129, A882, G222, G224, K220, K86, L170, M225, R263, S130, V132, and W117. A7 also established alkyl/π–alkyl interactions with F114 and I260 (Figure 4D).

### 3.6 Quantum chemical analysis

#### 3.6.1 Ligand optimization and validation

**FIGURE 5:**
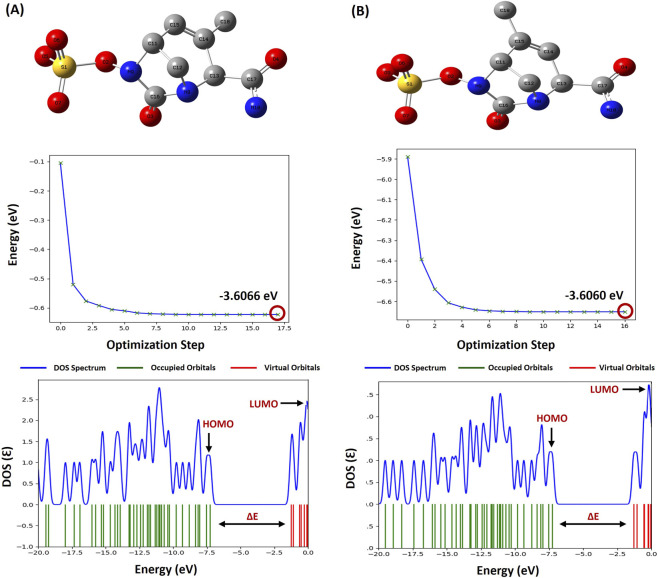
Ligand geometry optimization and electronic structure analysis: **(A)** durlobactam; **(B)** A7. Molecular structures are shown with colour-coded atoms; energy convergence plot (left) shows the optimization process while the density-of-states DOS plot (right) highlighted the HOMO, LUMO, and their energy gap.

Based on the binding energies and intermolecular interactions, A7 was prioritized, and, therefore, its chemical reactivity and stability profiles were adjudged by quantum chemical simulations upon comparing it with durlobactam. The optimized structures of durlobactam and A7 with their atomic numbering schemes, optimized trajectories highlighting the minimum energy (circled in red), and the DOS spectrum are illustrated in Figure 5. The structural parameters alterations such as bond length (Å), bond angles (°), and dihedral angles (°) involving crucial atoms [carbon (C), nitrogen (N), oxygen (O), and sulphur (S)] before and after optimization are presented in Supplementary Material SI3. Both the leads, durlobactam and A7, comprised of 29 atoms, including six oxygen, three nitrogen, eight carbon, 11 hydrogen, one sulphur, and a total of 144 electrons, respectively. The lowest energy conformers were determined to be −3.6066 eV for durlobactam and −3.6060 eV for A7, indicating that both ligands attained stability at approximately comparable energy levels (Figure 5). Based on the Mulliken population analysis, the graphical depiction of the composition of the molecular orbitals and the electronic contribution in forming chemical bonds were analysed using theDOS spectrum. The DOS spectra (blue) provided insights into the electron cloud in the quasi-degenerate energy state, where the occupied orbitals (donor) are represented by green lines and the unoccupied or virtual orbitals (acceptor) are shown in red. The positive DOS revealed the bonding interactions, whereas the zero (0.0) scores indicate their non-bonding characters. The energy gap (ΔE) between the occupied and virtual orbitals is represented as HOMO–LUMO energy levels.

#### 3.6.2 Frontier molecular orbital (FMO) and quantum theoretical speculations

**FIGURE 6:**
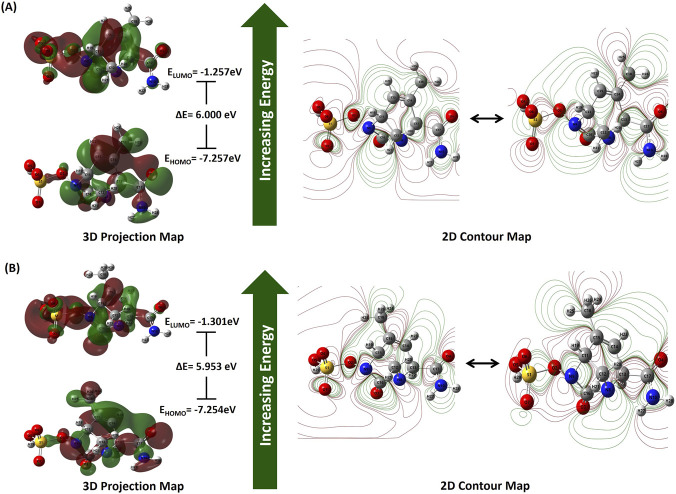
Frontier molecular orbital (FMO): DFT simulations highlighting the HOMO–LUMO profile in 3D projection and 2D contour map for **(A)** durlobactam and **(B)** A7.

FMO analyses for ligands were conducted primarily by the visualization of HOMO–LUMO iso-surfaces. HOMO corresponds to electron-filled orbitals and lone pairs capable of donating electrons, whereas LUMO represents the unoccupied or partially filled orbitals that can accept electrons. The energy gap (ΔE) indicating the ability of electron to shift from HOMO–LUMO energy levels is a critical parameter influencing the chemical reactivity, stability, electrical performance, and optical parameters of the ligand. The FMO of the ligands depicting the 3D projection and 2D contour maps are illustrated in Figure 6, where positive phases and negative phases are coloured in red and green, respectively. The energy gap $\left(\Delta E=E_{LUMO}-E_{HOMO}\right)$ of durlobactam and A7 was found to be 6.000 eV and 5.953 eV, respectively. A7 was noted to have lower energy gap (ΔE = 5.953 eV), ionisation energy (I = 7.254 eV), chemical hardness (η = 2.977 eV), and chemical potential (ρ = −4.278 eV), while the recorded dipole moment (µ = 1.4 Debye), electron affinity (A = 1.301 eV), electronegativity (χ = 4.278 eV), and chemical softness (δ = 0.336 eV) were higher than those of durlobactam (Table 3).

**TABLE 3:** FMO energies and global reactivity parameters calculated under the B3LYP/6–311G ++ (d,p) basis set for durlobactam and A7.

Global descriptors	Equation	Durlobactam	A7
Total energy (eV)	--	−3.6066	−3.6060
Dipole moment (µ) (debye)	μ=q*d	1.575	1.400
E_HOMO_ (eV)	Energy of HOMO	−7.257	−7.254
E_LUMO_ (eV)	Energy of LUMO	−1.257	−1.301
Energy gap (ΔE) (eV)	${\Delta E=E}_{LUMO}-E_{HOMO}$	6.000	5.953
Ionisation energy (I) (eV)	$I=-E_{HOMO}$	7.257	7.254
Electron affinity (A) (eV)	$A=-E_{LUMO}$	1.257	1.301
Electronegativity (χ) (eV)	$\chi =\frac{I+A}{2}$	4.257	4.278
Chemical potential (ρ) (eV)	$\rho =-\chi =-\frac{I+A}{2}$	−4.257	−4.278
Chemical hardness (η) (eV)	$\eta =\frac{I-A}{2}$	3.000	2.977
Chemical softness (δ) (eV^-1^)	$\delta =\frac{1}{\eta }=\frac{2}{I-A}$	0.333	0.336
Global electrophilicity index (ω) (eV)	$\omega =\frac{\rho^{2}}{2\eta }$	3.020	3.074
Global nucleophilicity index (N) (eV^-1^)	$N=\frac{I}{\omega }=\frac{2\eta }{\rho^{2}}$	0.331	0.325

q, magnitude of charge; d, distance between charges; HOMO, highest occupied molecular orbital; LUMO, lowest unoccupied molecular orbital; eV, electron volt.

#### 3.6.3 Electrostatic potential (ESP) analysis

**FIGURE 7:**
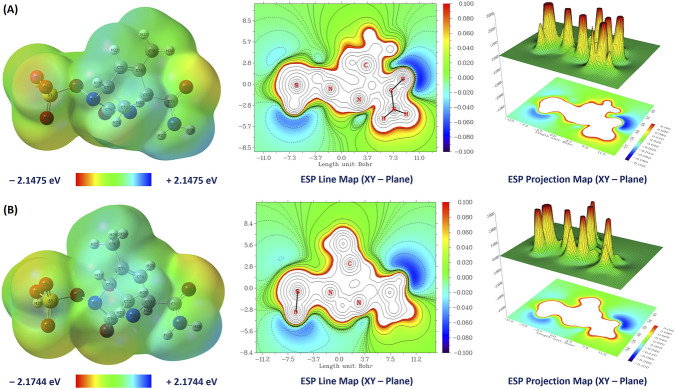
Molecular electrostatic potential: map highlighting the charge distribution of the molecule; line and projection map (XY planes) depicting the potential contours, while the 3D-plot highlights the reactive sites for **(A)** durlobactam and **(B)** A7.

The electronegative and electropositive localised centres of a molecule can be understood from the ESP analysis, which is crucial to determine the electrophilic or nucleophilic reaction that the molecule will undergo upon interacting with the target. The ESP molecular isosurface map represents electropositive centres that are illustrated in blue–green, while electronegative centres are illustrated in orange–red. A7 bears better ionisation potential and is more electronegative than its classical counterpart durlobactam, and it revealed slightly higher negative inductive effect owing to the double-bonded (π) and electronegative O4 and approximately O5–O6–O7 atoms, while the DBO ring at the centre of the molecule was electropositive. This explains the antibacterial potency of the DBO ring possessed by the ligands, which, being electron-deficient (electrophilic centres), experience a nucleophilic attack from the active S-X-X-K domain of the oxacillinase targets. Corresponding to it, the contour line map depicts blue hues adjacent to the electron-rich O-atoms, indicating the possible sites of nucleophilic attack with the electron-deficient centres of the targets (Figure 7).

#### 3.6.4 Natural bond orbitals (NBO) evaluation

The reactivity of the ligands was adjudged based on charge delocalization from bonding orbitals to their antibonding orbitals, which was determined based on NBO orbitals. The stability of the ligands was expressed as a function of stabilization energies (E^(2)^), which was calculated based on the Fock matrix derived from the second-perturbation theory, which was calculated using the formula:

$$E^{\left(2\right)}=\Delta E_{ij}=q_{i}\frac{{\left(F\left(i,j\right)\right)}^{2}}{E_{i}-E_{j}}.$$

Here, qi represents the donor orbital occupancy, F(i.j) represents the off diagonal NBO Fock matrix elements, and Ei and Ej represent the diagonal elements of the matrix.

In durlobactam and A7, the electronic transition from LP (N10) → π* (O4–C17) contributed the most to the molecule stability, with E^(2)^ stabilizing energies of 54.42 kcal/mol and 52.02 kcal/mol, followed by LP (O3) → σ* (N9–C16) with 30.63 kcal/mol and 30.87 kcal/mol; LP (O3) → σ* (N8–C16) with E^(2)^ of 27.35 kcal/mol and 27.41 kcal/mol; LP (O7) → σ* (S1–O2) with E^(2)^ of 26.23 kcal/mol and 26.55 kcal/mol; and LP (O4) → σ* (N10–C17) with E^(2)^ of 24.11 kcal/mol and 27.27 kcal/mol respectively. The 10 major electronic transitions tabulating the E^(2)^ energies, E(j)–E(i) and F(i.j) values, for all the studied ligands are tabulated in Table 4.

**TABLE 4:** Second-order perturbation theory analysis of Fock matrix in NBO basis of (a) durlobactam and (b) A7.

Donor NBO (i)		Acceptor NBO (j)		Stabilization energy E^(2)^ (kcal/mol)	Energy difference E(j) − E(i) (a.u.)	Polarized energy F (i,j) (a.u.)
Bond/Lone pair	Atoms	Antibonding	Atoms			
Durlobactam						
LP (1)	N10	BD* (2)	O4–C17	54.42	0.31	0.12
LP (2)	O3	BD* (1)	N9–C16	30.63	0.62	0.13
LP (2)	O3	BD* (1)	N8–C16	27.35	0.62	0.12
LP (3)	O7	BD* (1)	S1–O2	26.23	0.34	0.09
LP (2)	O4	BD* (1)	N10–C17	24.11	0.72	0.12
LP (3)	O6	BD* (1)	S1–O2	23.64	0.34	0.08
LP (1)	N9	BD* (2)	O3–C16	23.17	0.38	0.09
LP (2)	O4	BD* (1)	C13–C17	21.42	0.60	0.10
LP (3)	O6	BD* (1)	S1–O5	20.56	0.40	0.08
LP (3)	O7	BD* (1)	S1–O5	19.90	0.40	0.08
A7						
LP (1)	N10	BD* (2)	O4–C17	52.02	0.33	0.12
LP (2)	O3	BD* (1)	N9–C16	30.87	0.62	0.13
LP (2)	O3	BD* (1)	N8–C16	27.41	0.62	0.12
LP (3)	O7	BD* (1)	S1–O2	26.55	0.34	0.09
LP (2)	O4	BD* (1)	N10–C17	24.27	0.72	0.12
LP (1)	N9	BD* (2)	O3–C16	24.08	0.38	0.09
LP (3)	O6	BD* (1)	S1–O2	23.79	0.34	0.08
LP (2)	O4	BD* (1)	C13–C17	21.02	0.60	0.10
LP (3)	O6	BD* (1)	S1–O5	20.46	0.40	0.08
LP (3)	O7	BD* (1)	S1–O5	19.83	0.40	0.08

LP, lone pair; BD, bond pair.

* Antibonding.

E(j) − E(i), difference between donor (i) and acceptor (j) NBO orbitals; F (i,j), Fock matrix element between i and j NBO orbitals; E^(2)^, energy of hyper conjugative interactions (stabilizing energy). The figures in the bracket () represents the bond order.

#### 3.6.5 Electron localization function (ELF) and localized orbital locator (LOL)

**FIGURE 8:**
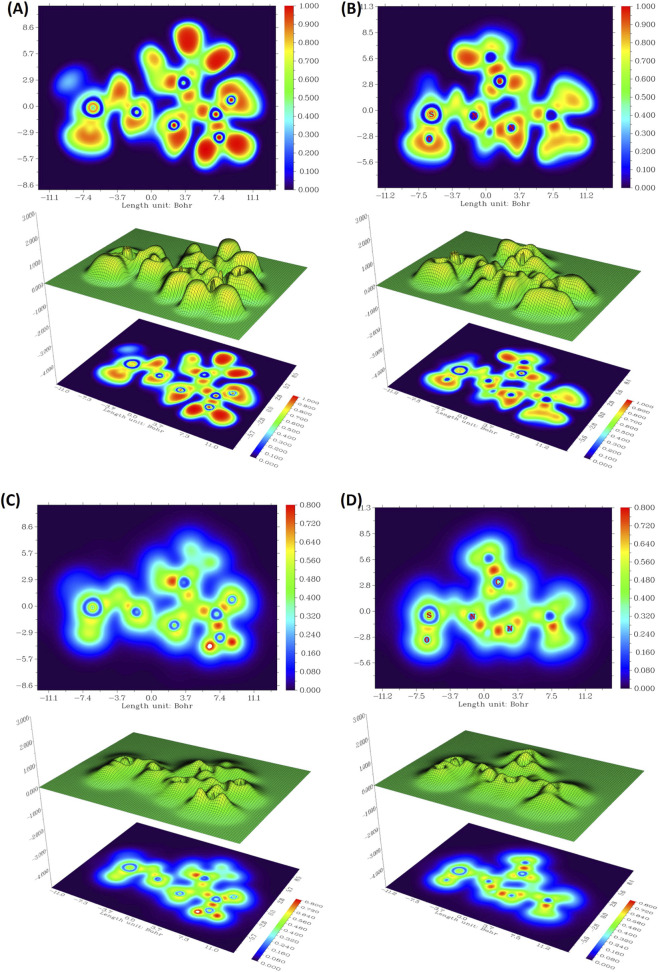
Electron localization function (ELF) and localized orbital locator (LOL) maps: ELF 2D contour plot showing electron localization–delocalization; 3D surface plot highlighting the regions of strong electron localization for **(A)** durlobactam and **(B)** A7; LOL 2D contour plot showing electron localization–delocalization; 3D surface plot highlighting regions of strong electron localization for **(C)** durlobactam and **(D)** A7.

The topological analyses of ELF and LOL are measures of covalent bond analysis, which complemented each other as both depends on kinetic energy density, revealing areas of high electron pairing potency. The ELF explains the electron pair density, whereas the LOL reveals gradients of localised orbitals. The interplay of localised and delocalised electrons is illustrated as relief and the projection map, as depicted in Figure 8. The ELF map ranges from 0.00 to 1.00, where the higher values (red zones) indicate electron localisation in bonding and non-bonding atoms, the yellow to green zone indicates medium localisation, and the blue zone (ELF <0.50) indicated electronic delocalisation. On the other hand, the LOL map ranges from 0.00 to 0.80 and provides more deeper insights indicating regions dominated by localised electrons (LOL >0.50) and domains governed by delocalised electrons (LOL <0.50).

#### 3.6.6 Assessment of the pharmacodynamics parameters through thermodynamics metrices

The thermodynamics parameters play a major role in the pharmacodynamics properties of ligands, including the reactivity and stability under varying temperatures. The change in thermodynamics parameters such as entropy (S), heat capacity (Cp), enthalpy (H), and Gibbs free energy (G) with increase in temperatures based on vibrational analysis are tabulated in Supplementary Material SI4. The thermodynamic properties of the ligands were assessed across a temperature range from 50 K to 1,000 K. It was observed that the entropy, heat capacity, and enthalpy increased with the increase in temperature, while Gibbs free energy gradually decreased. Table 5 shows the correlation between the thermodynamic parameters with respect to temperature, and it is expressed as fitting quadratic equations with the corresponding fitting factors (R^2^).

**TABLE 5:** Thermodynamic parameters represented with fitting quadratic equations and fitting factors (*R* ^2^) representing the entropy (S), heat capacity (Cp), enthalpy (H), and Gibbs free energy (G) with respect to increase in temperatures from 50 K to 1,000 K for durlobactam and A7.

Ligands	Fitting equations and fitting factors (*R* ^2^) for the thermodynamics parameters
Durlobactam	$S=54.465+0.249T-0.00006T^{2}\left(R^{2}=0.9994\right)$
	$C_{p}=3.1354+0.2286T-0.0001T^{2}\left(R^{2}=0.9995\right)$
	$H=131.29+0.0253T+0.00006T^{2}\left(R^{2}=0.9992\right)$
	$G=134.78-0.0684T-0.00009T^{2}\left(R^{2}=0.9999\right)$
A7	$S=54.926+0.2504T-0.0000{6T}^{2}\left(R^{2}=0.9998\right)$
	$C_{p}=3.57+0.2268T-0.0001T^{2}\left(R^{2}=0.9996\right)$
	$H=131.38+0.0254T+0.00006T^{2}\left(R^{2}=0.9993\right)$
	$G=134.89-0.0692T-0.00009T^{2}\left(R^{2}=1.9999\right)$

S, entropy; Cp, heat capacity; H, enthalpy; G, Gibbs free energy.

**FIGURE 9:**
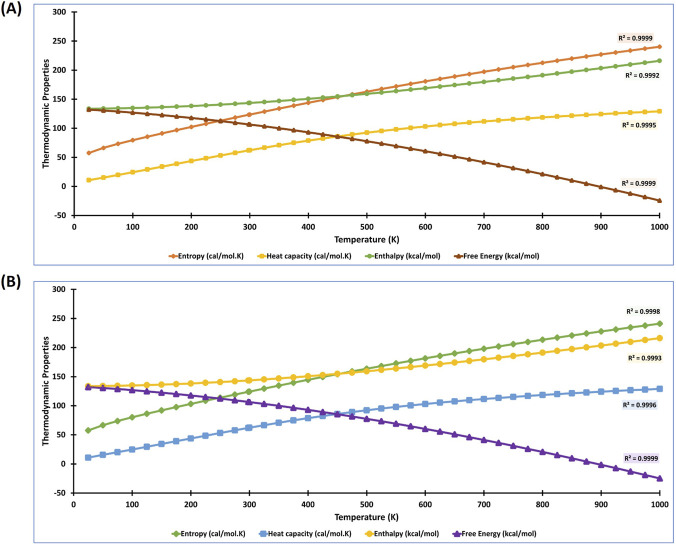
Pharmacodynamics (entropy, heat capacity, enthalpy, and free energy) fluctuations under varying temperatures (25 K–1,000 K) of **(A)** durlobactam and **(B)** A7.

The entropy of durlobactam steadily increased from 57.426 cal/mol.K at 25 K to 240.348 cal/mol.K at 1,000 K, along with an increase in heat capacity from 10.686 cal/mol.K to 129.098 cal/mol.K. Similarly, the enthalpy of durlobactam increased from 133.509 kcal/mol to 216.086 kcal/mol, while Gibbs free energy decreased from 132.073 kcal/mol to −24.262 kcal/mol, indicating enhanced pharmacodynamics properties at higher temperatures (Figure 9A). A7 displayed a similar pattern with the increase in entropy from 57.549 cal/mol.K to 241.057 cal/mol.K, increasing heat capacity from 10.823 cal/mol.K to 129.033 cal/mol.K, and increase in enthalpy from 133.579 kcal/mol to 216.145 kcal/mol. The Gibbs free energy initially measured 132.141 kcal/mol at 25 K and progressively decreased to −24.912 kcal/mol at 1,000 K, indicating increased reactivity with the increase in temperature (Figure 9B).

### 3.7 Stability assessment of the target–ligand complex

**FIGURE 10:**
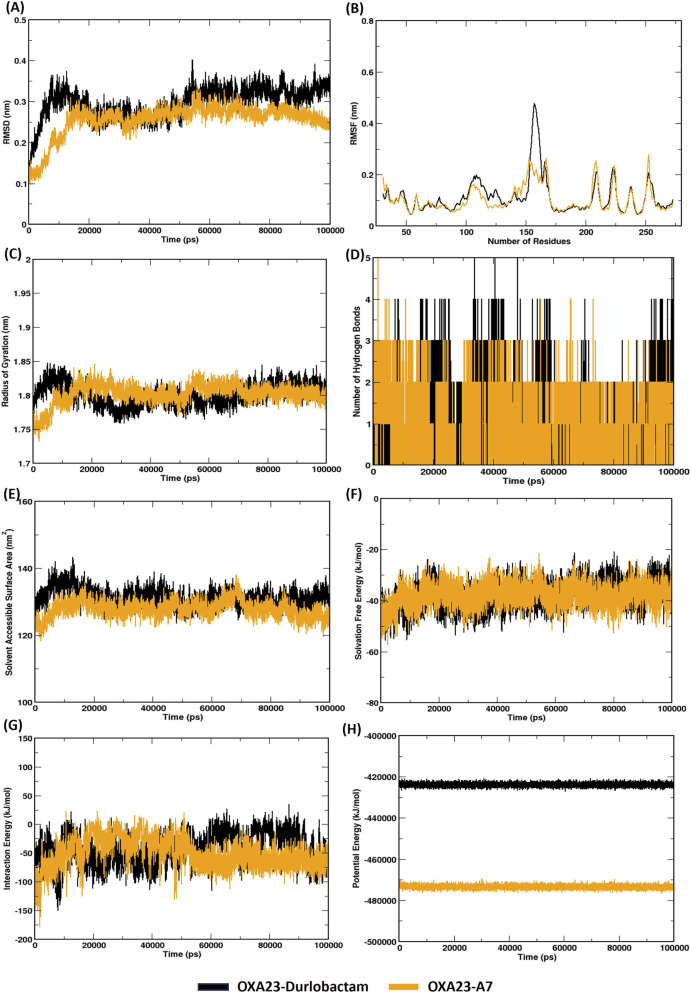
All-atom molecular dynamics simulation (MDS) of OXA23–durlobactam and OXA23–A7 complexes revealing trajectories such as **(A)** RMSD, **(B)** RMSF, **(C)** Rg plot, **(D)** hydrogen bonds, **(E)** SASA, **(F)** solvation free energy, **(G)** interaction energy, and **(H)** potential energy.

The complexes with better binding affinity and the favourable pharmacokinetic profiles were considered for stability assessment through MDS analysis. All the systems exhibited stable thermodynamic conditions during the two-phase equilibrations. The temperature of the system remained consistently at 300 K, indicating the effective thermal equilibration. The average pressure values fluctuated approximately at the reference of 1 bar, and the system densities converged within the range 1,028 kg/m^3^–1,039 kg/m^3^, revealing adequate pressure coupling and solvent equilibration, thus confirming that the systems were sufficiently relaxed prior to the MD run. Among the OXA23-bound ligands, durlobactam exhibited slightly higher RMSD of 0.31 ± 0.02 nm, while A7 showed comparatively lower deviation of 0.26 ± 0.02 nm (Figure 10A). The RMSF also followed a similar trend, with the OXA23–durlobactam complex displaying a slightly higher RMSF of 0.11 ± 0.01 nm compared to 0.10 ± 0.01 nm for the OXA23–A7 complex (Figure 10B). The Rg remained relatively stable across all the complexes, with the OXA23–durlobactam and OXA23–A7 complexes revealing an Rg of 1.80 ± 0.01 nm (Figure 10C), respectively. In the OXA23–durlobactam complex, a total of four hydrogen bonds were formed by durlobactam, but there was a substantial discontinuity in the hydrogen bond pattern throughout the simulation timeframe, while for OXA23–A7, a total of five hydrogen bonds were formed at the beginning, and at least two stable hydrogen bonds persisted throughout the simulation (Figure 10D). The compactness, indicated by the SASA, revealed that OXA23–durlobactam had slightly higher solvent exposure (131.27 ± 1.12 nm^2^) than OXA23–A7 (127.82 ± 0.88 nm^2^) (Figure 10E). The solvation free energy analysis indicated that OXA23–durlobactam and OXA23–A7 had ΔG_solv_ of −38.60 ± 1.93 kcal/mol and −37.95 ± 1.24 kcal/mol, respectively (Figure 10F). The interaction energy of the complexes was confirmed by obtaining the short-range Coulomb (Coul-SR) and short-range Lennard–Jones (LJ-SR) energy terms. The average Coul-SR values of OXA23–durlobactam and OXA23–A7 were −42.24 kJ/mol and −51.43 kJ/mol, respectively, whereas the LJ-SR energies of OXA23–durlobactam and OXA23–A7 were −70.04 kJ/mol and −90.06 kJ/mol, respectively (Figure 10G). Potential energy analysis revealed favourable energy profiles for both complexes; the OXA23–durlobactam complex showed a potential energy of −423,860.26 kJ/mol, and OXA23–A7 exhibited a lower and more favourable energy of −473408.06 kJ/mol (Figure 10H).

**FIGURE 11:**
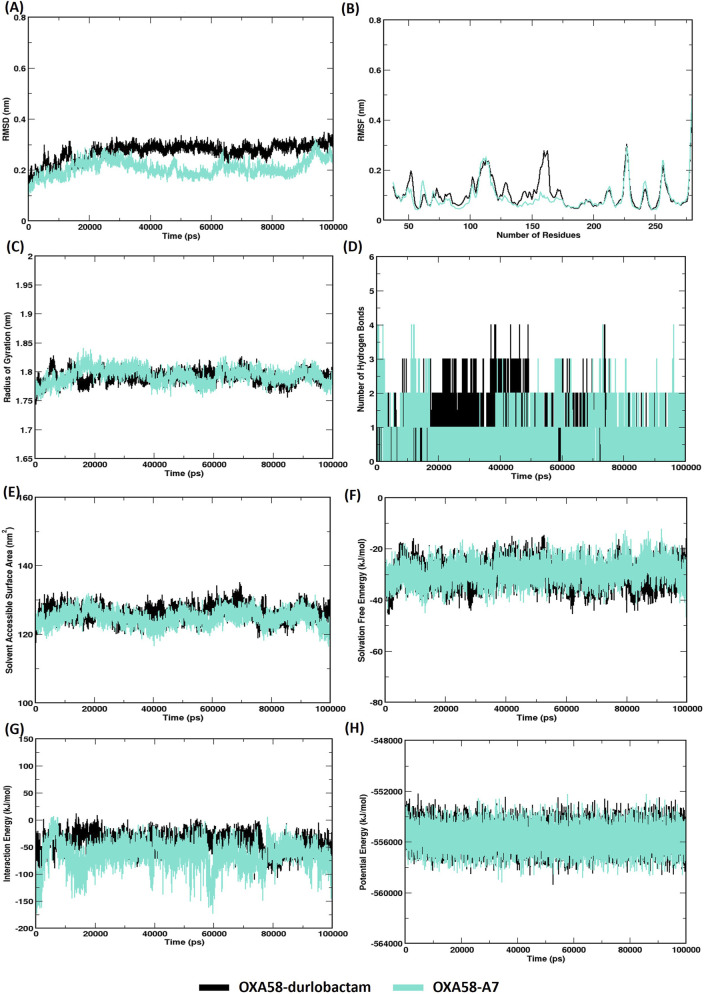
All-atom molecular dynamics simulation (MDS) of OXA58–durlobactam and OXA58–A7 complexes revealing trajectories such as **(A)** RMSD, **(B)** RMSF, **(C)** Rg plot, **(D)** hydrogen bonds, **(E)** SASA, **(F)** solvation free energy, **(G)** interaction energy, and **(H)** potential energy.

In case of OXA58, A7 exhibited superior stability with an RMSD of 0.21 ± 0.01 nm, while durlobactam showed a higher RMSD of 0.27 ± 0.01 nm (Figure 11A). The RMSF also reflected this pattern, with A7 showing less fluctuations (0.09 ± 0.01 nm) than durlobactam (0.11 ± 0.01 nm) (Figure 11B), while the Rg values remained constant across both complexes (1.79 ± 0.01 nm) (Figure 11C). For both OXA58-bound complexes, four hydrogen bonds were formed, but there was massive discontinuity in the hydrogen bond pattern during the simulation, with at least one bond maintained throughout the simulation timeframe (Figure 11D). The OXA58–durlobactam complex exhibited slightly higher SASA of 126.09 ± 0.97 nm^2^ than the OXA58–A7 complex, which had a solvent exposure of 124.76 ± 0.83 nm^2^ (Figure 11E). The solvation free energy analysis revealed that OXA58–durlobactam had slightly higher ΔG_solv_ of −30.41 ± 1.13 kcal/mol, while OXA58–A7 exhibited ΔG_solv_ of −29.60 ± 1.09 kcal/mol (Figure 11F). The average Coul-SR values of OXA58–durlobactam and OXA58–A7 were −43.72 kJ/mol and −65.16 kJ/mol, respectively, whereas the LJ-SR of OXA58–durlobactam and OXA58–A7 were −86.23 kJ/mol and −90.35 kJ/mol, respectively (Figure 11G). Potential energy analysis revealed that both the OXA58–durlobactam (−555595.35 kJ/mol) and OXA58–A7 (−555778.06 s kJ/mol) complexes exhibited favourable potential energy (Figure 11H).

**FIGURE 12:**
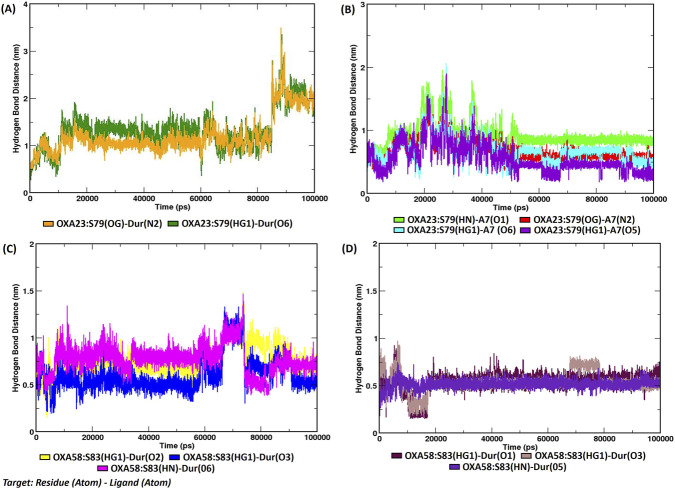
Hydrogen bond distance analysis: **(A)** OXA23–durlobactam complex; **(B)** OXA23–A7 complex; **(C)** OXA58–durlobactam complex; **(D)** OXA58–A7 complex.

The distance of the hydrogen bonds between the protein residues and the ligands were assesed using the donor–acceptor distance criterion. It revealed that A7 exhibited more proximal interactions with both the targets compared to durlobactam. The hydrogen bond interactions were represented as target: residue (atom)–ligand (atom) distance, where a larger distance indicates weaker interactions. It was observed that in the OXA23–durlobactam complex, durlobactam established comparatively weaker hydrogen bonds with OXA23, including OXA23:S79 (OG)–durlobactam (N2) – 1.20 ± 0.39 nm and OXA23:S79 (HG1)–durlobactam (O6) – 1.32 ± 0.40 nm (Figure 12A). In contrast, A7 exhibited multiple interactions with the catalytic S79 residue, including OXA23:S79 (HN)–A7(O1) – 0.92 ± 0.21 nm, OXA23:S79 (OG)–A7 (N2) – 0.68 ± 0.17 nm, OXA23:S79 (HG1)–A7 (O6) – 0.68 ± 0.20 nm, and OXA23:S79 (HG1)–A7 (O5) – 0.55 ± 0.22 nm (Figure 12B). The similar trend was observed in OXA58-bound complexes; in the OXA58–durlobactam complex, durlobactam formed weaker interactions with OXA58, including OXA58:S83(HG1)–durlobactam (O2) – 0.76 ± 0.15 nm, OXA58:S83 (HG1)–durlobactam (O3) – 0.60 ± 0.17 nm, and OXA58:S83 (HN)–durlobactam (O6) – 0.77 ± 0.14 nm (Figure 12C). In contrast A7 formed shorter interactions, including OXA58:S83 (HG1)–A7(O1) – 0.52 ± 0.10 nm, OXA58:S83 (HG1)–A7(O3) – 0.53 ± 0.10 nm, and OXA58:S83 (HN)–A7(O5) – 0.51 ± 0.04 nm (Figure 12D). This indicates the interaction proximity of A7 with the catalytic serine residues of OXA23 and OXA58.

### 3.8 Comparative binding evaluation of ligands across MDS-derived protein conformations

The conformational dependence of ligand-binding was assessed via molecular docking of 11 MDS-derived conformations of OXA23 and OXA58 targets (Table 6). For OXA23, both durlobactam and A7 showed moderate-to-strong binding across the conformations. A7 consistently exhibited more favourable binding profiles than durlobactam, with the strongest binding observed at 40 ns (−8.52 kcal/mol), 70 ns (−8.11 kcal/mol), 20 ns (−7.93 kcal/mol), 30 ns (−7.88 kcal/mol), 0 ns (−7.64 kcal/mol), 80 ns (−7.6 kcal/mol), 90 ns (−7.46 kcal/mol), 60 ns (−7.16 kcal/mol), and 10 (−7.03 kcal/mol), which are arranged in the decreasing order of the binding energies. In case of OXA58, both ligands demonstrated more variation in binding affinity. A7 exhibited favourable binding than durlobactam in the early and mid-simulation timeframes, particularly at 0 ns (−7.96 kcal/mol), 50 ns (−7.71 kcal/mol), 10 ns (−7.54 kcal/mol), 70 ns (−7.02 kcal/mol), 60 ns (−7.0 kcal/mol), 90 ns (−6.99 kcal/mol), 80 ns (−6.89 kcal/mol), and 20 ns (−6.30 kcal/mol), which are arranged in the decreasing order of the binding energies. A7 consistently performed better than durlobactam, exhibiting superior and more consistent binding across several simulation frames of both OXA23 and OXA58, thus highlighting its enhanced interaction potential within the catalytic sites of the targets. The intermolecular interaction profiles of all the docked complexes based on the target ensembles are illustrated in Supplementary Material S15.

**TABLE 6:** Simulation-based docking of ligands against MDS-derived protein conformations.

Complex		Binding energy (kcal/mol)	Inhibition constant (uM)	Complex		Binding energy (kcal/mol)	Inhibition constant (uM)
OXA23	0-Durlobactam	−7.37	3.96	OXA23	0-A7	**−7.64**	2.51
	10-Durlobactam	−6.89	8.95		10-A7	**−7.03**	7.03
	20-Durlobactam	−7.28	4.62		20-A7	**−7.93**	1.54
	30-Durlobactam	−6.97	7.74		30-A7	**−7.88**	1.68
	40-Durlobactam	−7.8	1.92		40-A7	**−8.52**	0.57
	50-Durlobactam	−7.24	4.93		50-A7	−7.19	5.37
	60-Durlobactam	−6.75	11.26		60-A7	**−7.16**	5.66
	70-Durlobactam	−6.75	11.23		70-A7	**−8.11**	1.14
	80-Durlobactam	−7.52	3.8		80-A7	**−7.6**	2.7
	90-Durlobactam	−7.07	6.52		90-A7	**−7.46**	3.38
	100-Durlobactam	−6.92	8.52		100-A7	−6.66	13.03
	*Average*	*−7.141*	*6.677*		*Average*	*−7.561*	*4.055*
OXA58	0-Durlobactam	−7.30	4.44	OXA58	0-A7	**−7.96**	1.47
	10-Durlobactam	−7.53	3.01		10-A7	**−7.54**	2.96
	20-Durlobactam	−6.11	33.47		20-A7	**−6.30**	24.02
	30-Durlobactam	−7.09	6.33		30-A7	−6.91	8.55
	40-Durlobactam	6.99	7.57		40-A7	−6.88	9.04
	50-Durlobactam	−7.62	7.57		50-A7	**−7.71**	2.25
	60-Durlobactam	−6.34	22.72		60-A7	**−7.00**	7.4
	70-Durlobactam	−6.95	8.06		70-A7	**−7.02**	7.19
	80-Durlobactam	−6.81	10.24		80-A7	**−6.89**	8.84
	90-Durlobactam	−6.75	11.23		90-A7	**−6.99**	7.48
	100-Durlobactam	−6.23	27.08		100-A7	−5.96	42.86
	*Average*	*−5.612*	*12.883*		*Average*	*−7.014*	*11.096*

Bold values indicate the superior binding affinity of A7 compared with durlobactam.

### 3.9 Essential dynamics and FEL analysis

**FIGURE 13:**
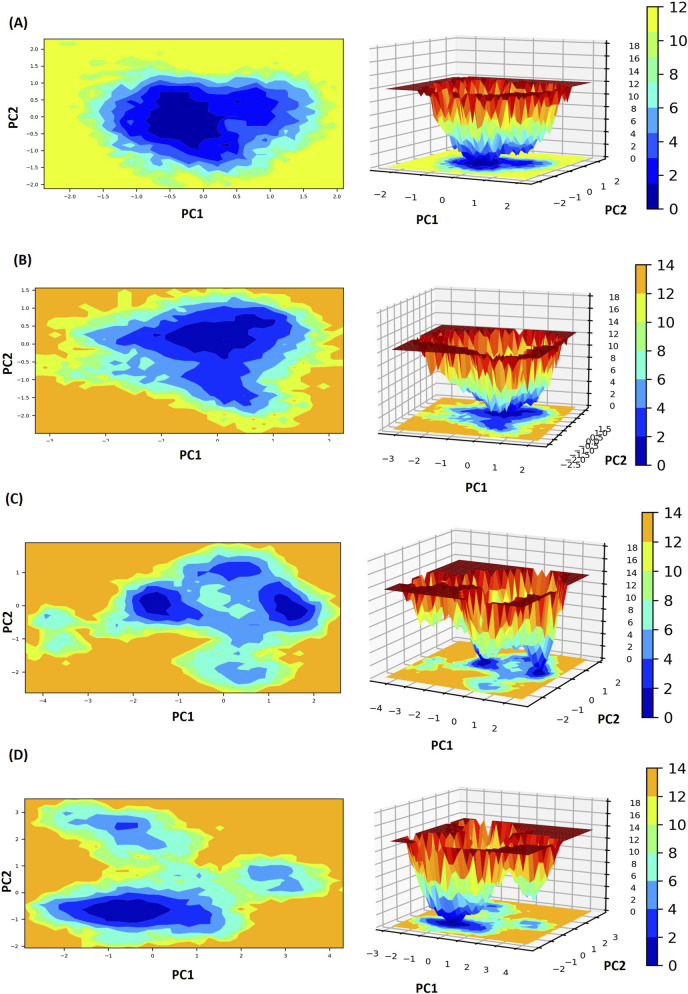
PCA–FEL 2D contour and 3D projection maps displayed as a function of PC1 and PC2 for: **(A)** OXA23–durlobactam; **(B)** OXA23–A7; **(C)** OXA58–durlobactam; **(D)** OXA58–A7.

The conformational flexibility and overall strenuous motions of the complexes were evaluated using PCA and FEL plots. The dynamic behaviour of protein in response to ligand binding, its corresponding fluctuations, and the dominant motions are captured by the eigenvalues and eigenvectors. Eigenvectors derived from the covariance matrix (principal components) were projected on the protein backbone depicting the residual motions of the system. The trace of covariance matrix, which indicates the overall conformational space explored by the system for OXA23–durlobactam (Figure 13A), OXA23–A7 (Figure 13B), OXA58–durlobactam (Figure 13C), and OXA58–A7 (Figure 13D) were computed to be 6.45309 nm^2^, 9.68126 nm^2^, 10.7278 nm^2^, and 8.59038 nm^2^, respectively. The two principal components with cosine content under 0.2 were selected to generate the FEL plot. The maps were constructed using the reaction coordinates from the selected principal components, which is represented as Gibbs free energy. The cluster basin with the lowest free energy is illustrated in dark-to-light blue, which are considered the stable protein conformer, validating the stability of the complexes. The Gibbs free energies for the most conformational stages ranged from 0 kJ/mol–11.9 kJ/mol (OXA23–durlobactam), 0 kJ/mol–12.3 kJ/mol (OXA23–A7), 0 kJ/mol–13.6 kJ/mol (OXA58–durlobactam), and 0 kJ/mol–13.5 kJ/mol (OXA58–A7), validating the thermodynamic stability of the complexes throughout the simulation.

### 3.10 Binding free energy calculations

**FIGURE 14:**
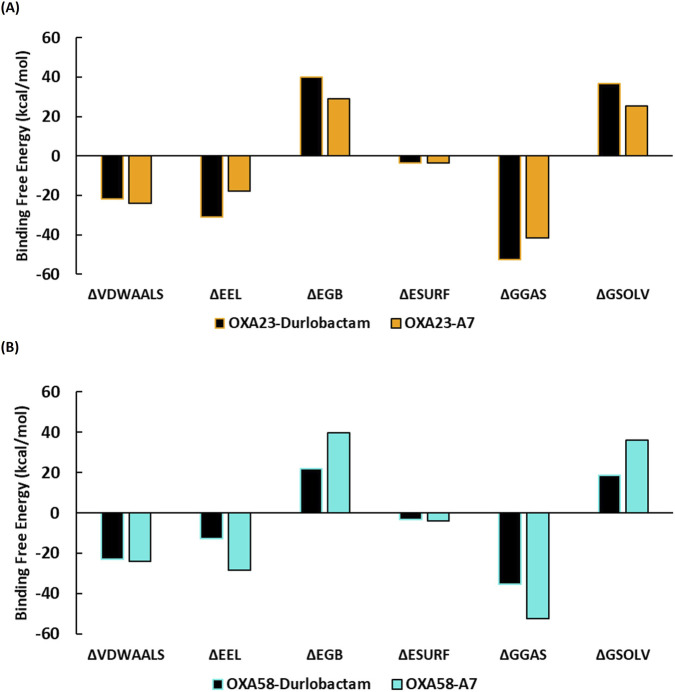
MMGBSA binding-free energy calculations of **(A)** OXA23–durlobactam; OXA23–A7; **(B)** OXA58–durlobactam; OXA58–A7.

The MM-GBSA was conducted over the 100-ns simulation timeframe to evaluate the binding free energies of the complexes. The energy components calculated included van der Waals molecular mechanics energy (ΔG_*VDWAALS*_), electrostatic molecular mechanics energy (ΔE_*EL*_), polar contribution to the solvation energy (ΔE_*GB*_), solvent accessible surface area (ΔE_*SURF*_), total gas-phase molecular mechanics energy (ΔG_*GAS*_), and total solvation energy (ΔG_*SOLV*_) (Table 7). The calculated ΔG_*Binding*_ revealed that the OXA58–durlobactam complex exhibited the most favourable ΔG_*Binding*_ of −16.69 kcal/mol, followed by OXA58–A7 (−16.24 kcal/mol), OXA23–A7 (−16.22 kcal/mol), and OXA23–durlobactam (−15.89 kcal/mol), respectively (Figure 14).

**TABLE 7:** Binding free energy calculations of the protein–ligand complexes.

Complex	ΔG_*VDWAALS*_	ΔE_*EL*_	ΔE_*GB*_	ΔE_*SURF*_	ΔG_*GAS*_	ΔG_*SOLV*_	ΔG_*Binding*_
OXA23–Durlobactam	−21.68 ± 4.2	−30.95 ± 12.79	40.14 ± 12.5	−3.39 ± 0.67	−52.63 ± 15.86	36.74 ± 11.94	−15.89 ± 4.97
OXA23–A7	−23.86 ± 5.19	−17.83 ± 8.11	29.01 ± 6.83	−3.54 ± 0.73	−41.69 ± 9.93	25.47 ± 6.54	−16.22 ± 5.4
OXA58–Durlobactam	−22.72 ± 3.02	−12.65 ± 6.2	21.91 ± 5.35	−3.23 ± 0.36	−35.37 ± 6.99	18.68 ± 5.2	−16.69 ± 3.21
OXA58–A7	−24.04 ± 3	−28.18 ± 8.91	39.76 ± 7.42	−3.78 ± 0.36	−52.22 ± 9.36	35.98 ± 7.35	−16.24 ± 3.51

ΔG_VDWAALS_, van der Waals molecular mechanics energy; ΔE_EL_, electrostatic molecular mechanics energy; ΔE_GB_, polar contribution to the solvation energy; ΔE_SURF_, solvent-accessible surface area; ΔG_GAS_, total gas-phase molecular mechanics energy; ΔG_SOLV_, total solvation energy; ΔG_Binding_, total relative binding energy.

## 4 Discussion

The βLIs such as durlobactam have emerged as clinically relevant scaffolds in recent years, especially against XDR pathogens such as *A. baumannii* (Moussa et al., 2023; Vijayakumar et al., 2024; Mazumder et al., 2026). Durlobactam demonstrated significant potency against class-D β-lactamases that are major contributors to β-lactam resistance in clinical isolates (Granata et al., 2022). It synergizes with β-lactam antibiotics, restoring their efficacy against the resistant strains. However, recent reports indicate the emergence of resistance towards durlobactam, particularly in the strains producing oxacillinases, which limits its long-term clinical utility (Principe et al., 2022). Therefore, in the current study, a library of durlobactam analogues was constructed to explore their computational binding efficacy and structural stability against clinical strains of *A. baumannii* that produce oxacillinases . The resistance profiling of all the 28 isolates and the predominance of *bla*_*OXA23*_ and *bla*_*OXA58*_ expression among the isolates supports the rationale of targeting class-D β-lactamases. The utilization of previously validated receptors for OXA23 and OXA58 further ensured the relevance of semi-empirical molecular docking and molecular dynamics simulations. The ligand-based virtual screening explored a set of analogues with high structural similarity to durlobactam. Furthermore, shortlisting of the analogue based on the Tanimoto coefficient allowed the selection of potential leads that are likely to retain the desired pharmacophoric interactions that are essential for the inhibitory activity, thus, in-turn, strengthening the reliability of the lead selection. The subsequent ADMET profiling of the shortlisted leads revealed that the physicochemical and pharmacokinetic descriptors remained within the drug-likeness ranges, aligning with the benchmarks established, such as Lipinski’s and Veber’s criteria. Furthermore, the absence of the toxicity risk and favourable absorption and metabolic profiles supports their computational drug-likeliness and prioritization for further evaluation.

The comparative molecular docking and interaction analysis of the shortlisted analogues highlighted the promising potential of a 4-methyl-substituted durlobactam analogue (A7) against OXA23 and OXA58 targets. The binding efficacy of A7, as inferred from comparatively lower docking scores across both the drug-targets, underscores its structural compatibility and target affinity. The inhibitory potential of A7 can be rationalized by its effective engagement within the conserved S-X-X-K catalytic motif, which is the hallmark for acylation and β-lactam hydrolysis of the class-D β-lactamases. A7 has formed stable hydrogen bonds with residues involved in the S-X-X-K domain of OXA23 and OXA58 targets, including S79, K82 in OXA23, and S83 in OXA58, which are a part of active serine responsible for nucleophilic attack during catalysis. This interaction with the active-site serine residue indicates that A7 may competitively occupy the catalytic groove, thereby obstructing the substrate access and inhibiting the enzymatic activity, as inferred from current computational assessments, which require further experimental validations (Naha et al., 2021).

The quantum chemical simulations carried out for durlobactam and its analogue A7 revealed minor differences in bond lengths, bond angles, and dihedrals with reduced bond lengths in A7 due to the presence of electronegative atoms in S1–O5, S1–O6, S1–O7, O2–N9, O3–C16, O4–C17, N8–C16, N9–C16, and N10–C17 (Supplementary Material S13). Similarly, bond angle constriction due to electronegative atoms and relaxation in dihedrals were also seen to minimize the steric clashes and orbital overlaps in the initial conformers, thus making the molecules gain the lowest energy and stability. The electronic transitions from the NBO analysis (Table 4) revealed that the delocalization of LP → σ*/π* electrons played crucial roles in the stability of both durlobactam and A7. Out of the 10 crucial electronic shifts governing the stability to the molecules, it can be clearly observed that the majority of the transitions for A7 recorded higher stabilization energies than its classical counterpart for the same atom-pairs, indicating that these transitions imparted better stability to the molecule. Envisaging on the reactivity of the studied ligands, FMO analysis displayed a slightly smaller ΔG (∼0.05 eV) for A7 than for durlobactam. Given that the magnitude of the difference is small, the reduced energy gap is considered as a supportive electronic feature that may subtly influence the electronic and reactivity profile of A7 in conjunction with all other electronic descriptors and wavefunction parameters, which is in consistency with the previously reported studies (Abrosimov and Moosmann, 2024; Hadigheh Rezvan et al., 2026). The DBO ring functions as the hub centre, and being electron deficient (blue-green hue in ESP map), it is an ideal centre for nucleophilic attacks by the active serine (S79 and S83) of the S-X-X-K-conserved domains of OXA23 and OXA58, respectively. The higher global electrophilicity index of A7 makes it a better electrophile than durlobactam, where the active serine has better affinity to form an irreversible complex upon attacking the carbonyl carbon, thus acylating the enzyme and simultaneously restoring the β-lactam antibiotics by blocking the active site based on the computational analysis; this finding requires further validation through enzymatic assays. The oxyanion groove stabilizes the negatively charged O-atoms (oxyanion) of the tetrahedral intermediate formed during the interaction through transient van der Waals and other NCIs seen in the intermolecular interactions. The lower chemical potential of A7 indicates greater thermodynamic stability, while the greater electronegativity and chemical softness indicate the electron delocalisation potency and reactivity of the molecule.

From Figure 8 depicting the ELF and LOL maps, it is evident that the H-atoms revealed high ELF (red zones), indicating the presence of localised electrons, while regions of delocalised electrons (blue zones) were governed by the presence of lone pairs and covalent bonding, and the regions around O, N, S, and C atoms were observed in both durlobactam (Figure 8A) and A7 (Figure 8B). Simultaneously, high electron density was observed around the H-atoms in LOL maps, which were so dense that white centres were seen forming because it surpassed the upper threshold of 0.80. Due to the presence of delocalised electrons around O, N, S, and C atoms for durlobactam (Figure 8C) and A7 (Figure 8D), LOL maps revealed blue colouration, indicating covalent interaction and delocalised electronic regions around them. Upon comparing both, A7 exhibited higher electronic delocalization, as indicated by strong blue-coloured zones and milder 3D peaks, which signified low electron pair potential and better stability of the ligand.

The theoretical harmonic frequencies rely on the vibrational analysis such as entropy, heat capacity, enthalpy, and Gibbs free energy, which are used to infer the pharmacodynamics of the ligands. The pharmacodynamics that is expressed in terms of standard thermodynamic terms provides insights on the molecule stability and spontaneity under varying temperatures (Figure 9) (Tarika et al., 2021). Increase in entropy indicates greater flexibility and molecular disorderness, which indicate the dynamic nature and enhanced interaction ability of the ligands with the interacting targets, while increased enthalpy is typical in complex molecules requiring more energy for maintaining their structural integrity under increased temperature. Similarly, increase in heat capacity reflects increased degrees of freedom in the molecule, which contribute to their stability and adaptability under varying biological conditions experienced by the body during inflammation and fever. A sharp decrease in Gibbs free energy indicates the spontaneity of the molecule, making the ligands thermodynamically more favourable and reactive. From all the four pharmacodynamic parameters, A7 revealed slightly better parameters than durlobactam, indicating favourable conformational and energetic features, and it engages in better inhibitory interactions, reflecting its better lead characteristics than durlobactam under varying temperatures under computational conditions (Raja et al., 2017; Arulaabaranam et al., 2021; Tarika et al., 2021).

The MD stability metrices indicated that A7-bound complexes exhibited dynamically and energetically stable complexes than those bound with durlobactam with both OXA23 and OXA58 β-lactamases (Figures 10, 11). The dynamic parameters of A7 consistently revealed lower RMSD and RMSF, indicating more conformational restraints and reduced atomic flexibility. The lower backbone deviations of A7-bound complexes revealed that the introduction of the ligand reduced the local flexibility and structural perturbations around the active site, facilitating the conformationally constrained binding state of the oxacillinases. In contrast, the marginally higher fluctuations observed for the durlobactam complexes indicated less efficient stabilization of the catalytic site. Additionally, the Rg remained stable throughout the complexes, but the slightly lower SASA of A7 indicated better compactness and burial within the core of the protein. The interaction energy decomposition of A7 indicated stronger hydrophobic and electrostatic contacts within the catalytic residue, which was reflected by the more favourable Coulombic and van der Waals interactions. The hydrogen bonding patterns provide a mechanistic insight into the stability of the complex. While the initial numbers of hydrogen bonds formed by both the ligands are comparable, A7 showed more persistent hydrogen bonds throughout the simulation. In the A7-bound complex not only formed a greater number of hydrogen bonds but also maintained the persistently shorter donor–acceptor contacts. This interaction is expected to contribute to the strong anchorage of ligand molecule within the active site groove, which may lead to the effective inhibition by forming the catalytically incompetent state of the enzyme. Discontinuous hydrogen bond formation was observed in durlobactam-bound complexes, which implies transient and relatively longer contact with active serine, which may weaken the effective inhibition. The energy profiles of the complexes further supported A7 as a superior inhibitory lead molecule. The interaction energies revealed more favourable short-range electrostatic and van der Waals energies for A7-bound complexes and indicated that A7 formed stronger contacts within the active-site binding cleft. Notably, in A7-bound complexes, the potential energy profiles were consistently more negative, which is consistent with the increased thermodynamic stability of the complex. Furthermore, the solvation free analysis showed better hydration dynamics for A7, supporting its compatibility in the physiological environment. The dynamics and energetics collectively prioritise A7 as a computationally favourable analogue over durlobactam (Naha et al., 2022).

The stability of the complexes during the MDS was governed by a comprehensive network of non-covalent interactions within the active site. The persistent hydrogen bonds and C–H bonds between the ligand and the key catalytic residues are expected to contribute to maintaining stable binding throughout the course of the simulation. This helps in restraining the ligand within the dynamically fluctuating catalytic domain of the target. Similarly, the van der Waals interactions are expected to play a crucial role in ensuring steric complementarity and compact packing of the ligand within the hydrophobic binding pocket. The alkyl and pi–alkyl interactions may further anchor and stabilize the ligand within the non-polar catalytic residues, thereby limiting the translational and rotational motions. Additionally, the pi–sulphur interactions are anticipated to provide the essential electronic stabilization, especially the interaction involving sulphur-containing residues. Collectively, these interactions are expected to minimize ligand displacement under dynamic conditions, supporting a stably bound conformation of the complex, which is important for the inhibitory potential of the ligand. The comparative binding across the MDS-derived protein conformations was used to further evaluate the stability, and A7 consistently showed more efficient and more sustained binding affinities. Durlobactam, on the other hand, demonstrated lower and varied affinities towards the targets, indicating weaker adaptability to conformational variations. A7 exhibited an average binding energy of −7.56 kcal/mol and −7.01 kcal/mol with OXA23 and OXA58, respectively. A7 showed remarkable docking scores over a range of timeframes, including the mid-simulation windows, despite increasing the variability, indicating better compatibility with the protein’s conformational ensembles. This dynamic competence highlights the pharmacological significance of A7 by demonstrating its ability to adapt and stabilize within the fluctuating targets. MM-GBSA-based binding free energy calculation provided quantitative insights into the thermodynamic stability of ligand-bound complexes across the 100-ns simulation timeframe, which further corroborated the findings (Figure 14). The overall stability of both the ligands in OXA23 and OXA58 targets was highlighted by the close clustering of the binding free energies throughout the complex. A7 consistently exhibited stronger van der Waals interactions, indicating enhanced hydrophobic contacts within the binding cleft. The ΔE_*EL*_ electrostatic contributions were more pronounced in the OXA23–durlobactam complex, but this gain was offset by ΔG_*SOLV*_ higher solvation penalty, leading to less favourable net binding energy. In contrast, A7 maintained a balance between gas-phase interactions and solvation energies, especially in the case of OXA58, where it achieved a ΔG_*Binding*_ comparable to that of durlobactam supported by efficient electrostatic and hydrophobic stabilization rather than through energetic superiority.

To complement the structural and energetic analysis, essential dynamics was utilized to characterise large-scale motions and conformational adaptations of complexes. The PCA revealed the distinct patterns of motions influenced by ligand binding, with trace value of covariance matrix reflecting the extent of the conformational space explored. The OXA58–durlobactam complex exhibited higher trace values, indicating the overall flexibility, while A7-bound complexes showed more restricted motion. The A7-bound complexes also consistently mapped stable energy cluster basins in FEL, as evidenced by the well-defined low-energy cluster basins observed across the simulations. The Gibbs free energy landscape across all the complexes remained within a stable range (0 kJ/mol–13 kJ/mol), supporting the thermodynamic favourability of the conformational states (George et al., 2025a). While A7 is identified in the ChEMBL database, computational insights into clinically derived oxacillinases have not been reported previously. In the current study, multiple *in silico* assessments that were utilized contribute to the insights into the behaviour of the ligand within the catalytic domain of β-lactamases, enabling the evaluation of its characteristics, rather than the direct inference of biological inhibition. Molecular docking was used to assess the initial binding feasibility by analysing the ligand orientation, steric complementarity, and interactions within the key active-site residues. The MD metrices further characterized the time-dependent behaviour of the complexes, allowing the assessment of ligand interactions with the active-site under dynamic, solvated, and near physiological environment (Mohapatra et al., 2023). The persistent nature of non-covalent interactions may contribute to the stabilization of ligand positioning, whereas the binding free energy calculations provides a relative thermodynamic favourability of the complex formation. The PCA-FEL delineates the dominant collective motions and conformations of the system, allowing the assessment of ligand biding with energetically stable and functionally relevant ensembles. The convergence of these computational outputs provides a systematic framework for the evaluation of the behaviour of A7 within the catalytic domain of OXA23 and OXA58. Importantly, these present findings do not demonstrate enzymatic or biological inhibition. Instead, the observed computational trends support the probability of stable active-site accommodation of A7 under the dynamic conditions, which is a pre requisite for β-lactamase inhibition. By explicitly linking the computational outputs to functional aspects, the study strengthens the biological interpretation of the findings while acknowledging the need for experimental validation to confirm the inhibitory function. This seamless alignment underscores A7, a 4-methyl-substituted analogue of durlobactam, chemically known as (2s,5r)-4-Methyl-7-Oxo-6-(Sulfooxy)-1,6-Diazabicyclo [3.2.1]oct-3-Ene-2-Carboxamide, strictly as a computationally evaluated ligand against oxacillinases of nosocomial XDR *A. baumannii*, providing a strong computational benchmark for subsequent experimental validations.

## 5 Conclusion

The comprehensive computational analysis identifies that the durlobactam analogue A7 is a promising exploratory βLI against the MDR and XDR *A. baumannii*. Pharmacokinetic profiling, molecular docking, quantum chemical analysis, molecular dynamics, and essential dynamics consistently demonstrated the stable binding, favourable reactivity, and conformational adaptability of A7 over durlobactam. Particularly, these parameters collectively underscore the importance of the stability and persistence of interactions, along with the thermodynamic favourability of A7. These findings highlight the potential of the 4-methyl-substituted durlobactam analogue against oxacillinases of nosocomial *A. baumannii.* While the present study is computational in nature, the observations offer a strong primary foundation for further *in vitro* and *in vivo* experimental validations. Future assessments should focus on cytotoxicity evaluations and enzymatic inhibition assays to substantiate the translational suitability. Collectively, this contributes to the understanding of the structure-based drug design through the lenses of cheminformatics and structural bioinformatics in the rational identification of durlobactam analogues to mitigate AMR.

## Data Availability

The original contributions presented in the study are included in the article/Supplementary Material further enquiries can be directed to the corresponding author.
