# Supplementary figures and images for: A 4-methyl-substituted durlobactam analogue as a potential class-D oxacillinase inhibitor in Acinetobacter baumannii—an in silico study
Source: Front Bioinform. 2026 May 26;6:1790411. doi: 10.3389/fbinf.2026.1790411 (PMC13246729; doi:10.3389/fbinf.2026.1790411)

**Supplementary Information SI5**


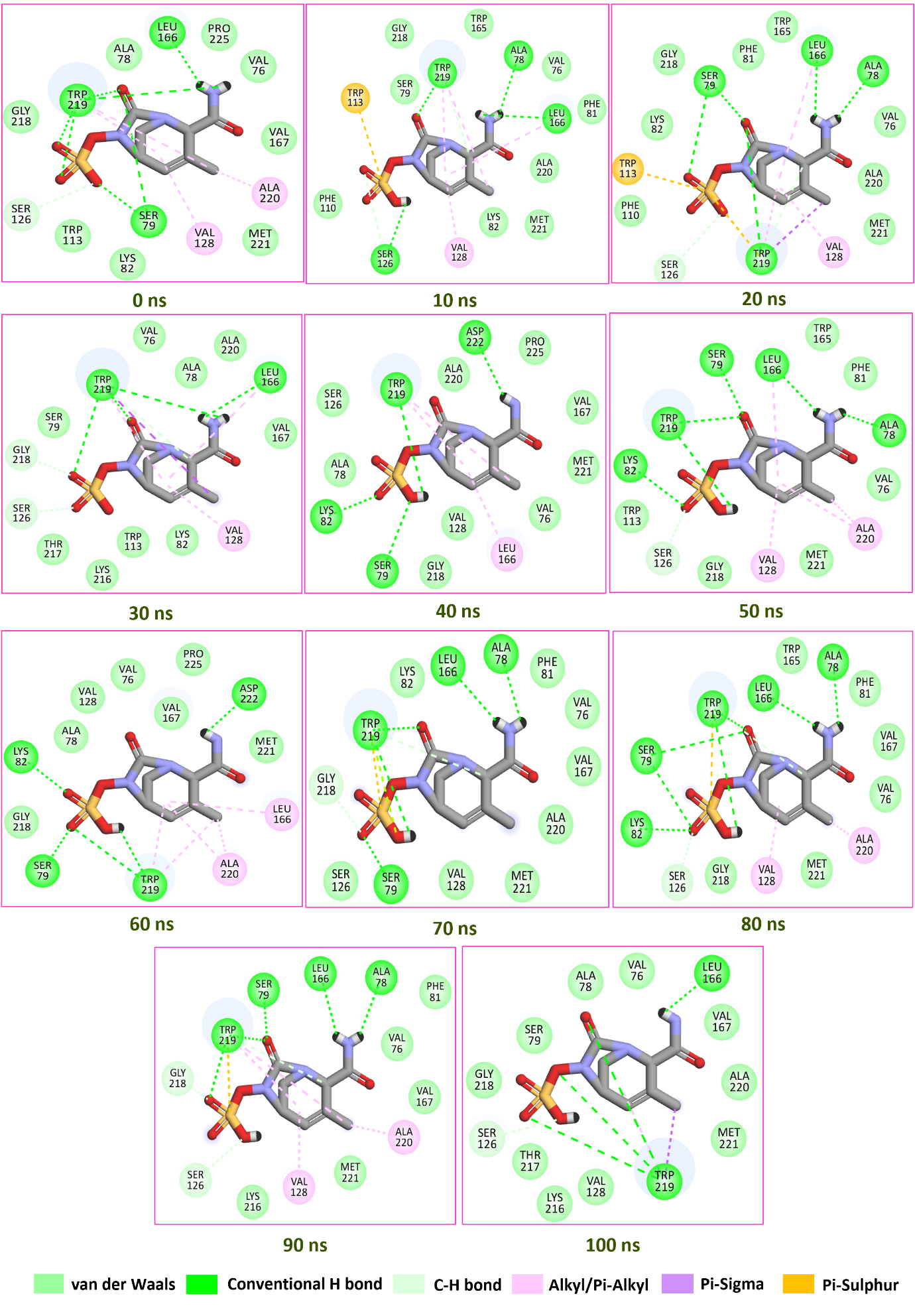


**OXA23-Durlobactam**


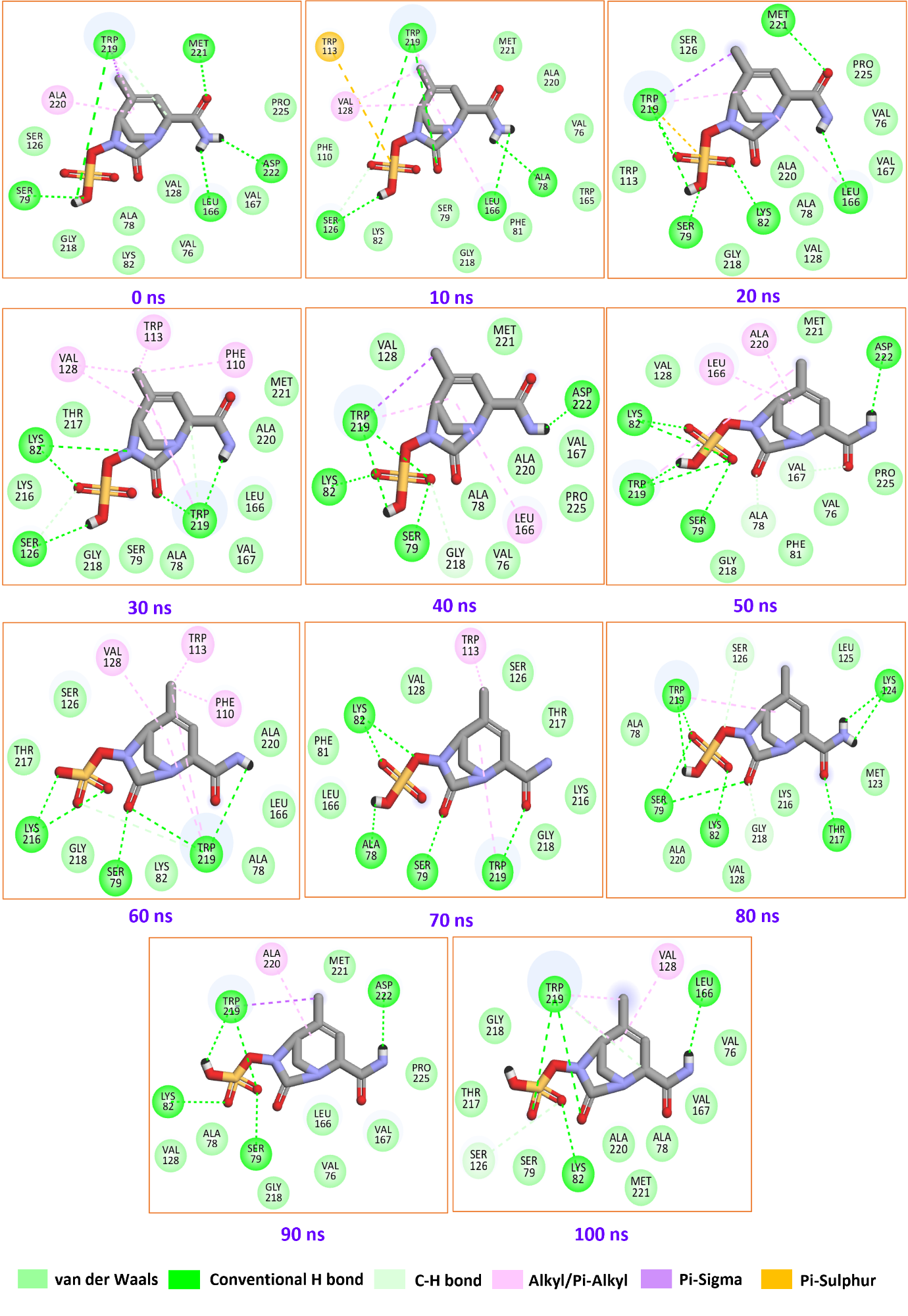


**OXA23-A7**

**
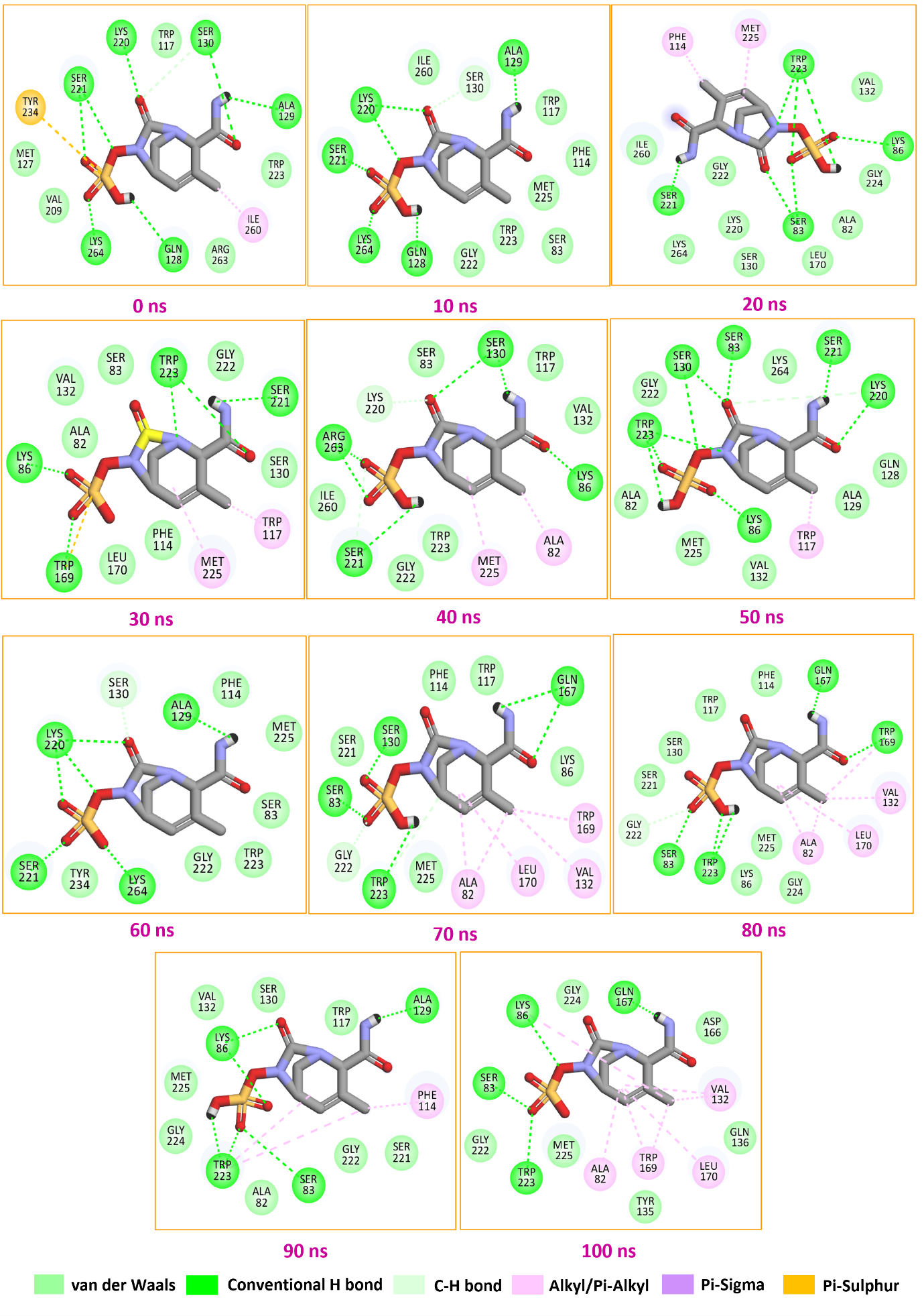
**

**OXA58-Durlobactam**

**
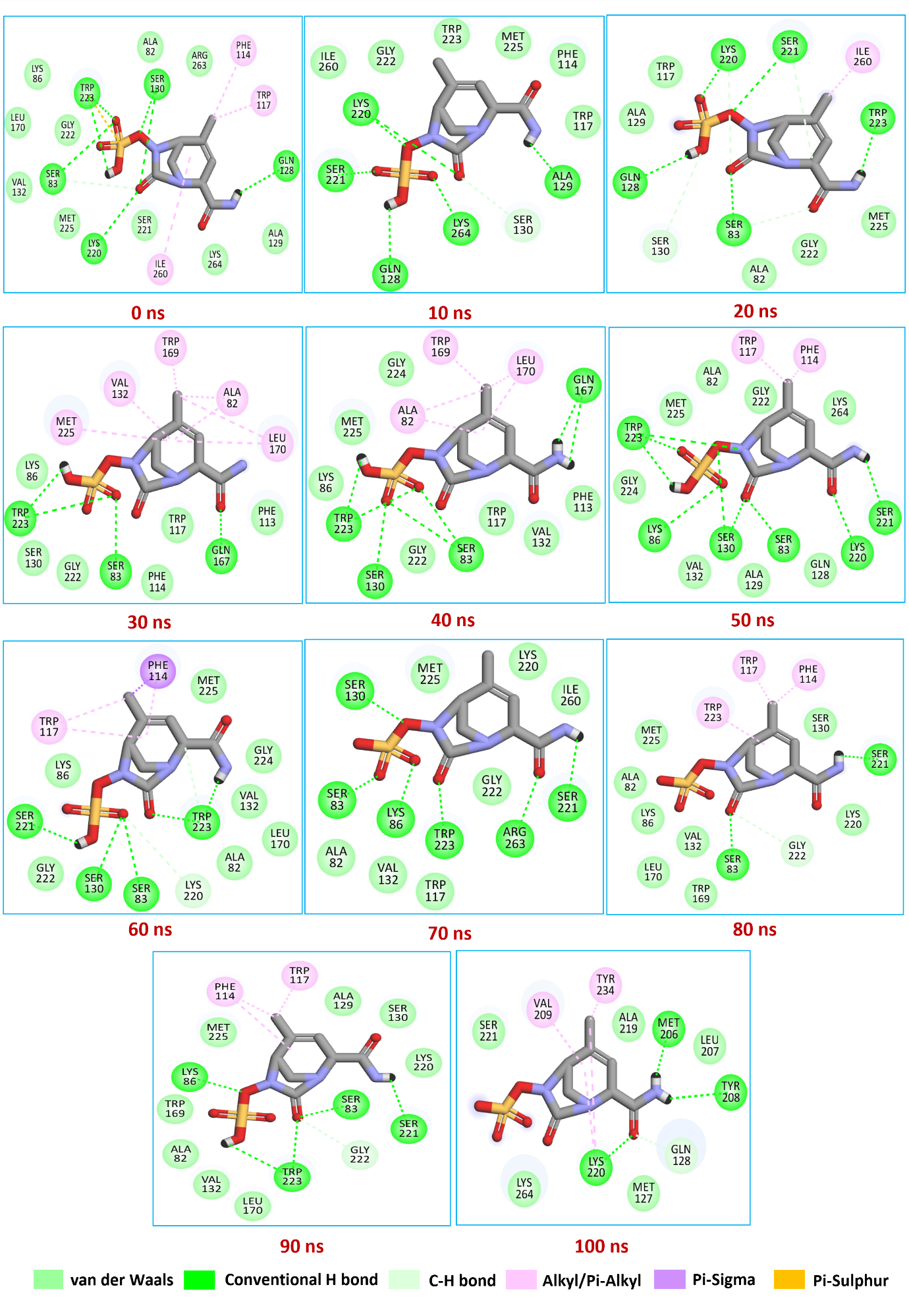
**

**OXA23-A7**

Supplement: Supplementary file 2 [file Table5.docx]
