# Supplementary material for: A 4-methyl-substituted durlobactam analogue as a potential class-D oxacillinase inhibitor in Acinetobacter baumannii—an in silico study
Source: Front Bioinform. 2026 May 26;6:1790411. doi: 10.3389/fbinf.2026.1790411 (PMC13246729; doi:10.3389/fbinf.2026.1790411)
Supplement: Supplementary file 4 [file Table3.docx]

**Supplementary Information SI3**

**Supplementary Information SI3:** Bond lengths, bond angles and dihedral angles before and after DFT optimization calculated under B3LYP/6–311++G (d,p) basis set for **(a)** Durlobactam, **(b)** Analogue 7

| **Bond Length** | **Angstrom (Å)** | | **Bond Angle** | **Degree (°)** | | **Dihedral Angle** | **Degree (°)** | |
| --- | --- | --- | --- | --- | --- | --- | --- | --- |
|  | **B.O.** | **A.O.** |  | **B.O.** | **A.O.** |  | **B.O.** | **A.O.** |
| **(a) Durlobactam** | | | | | | | | |
| S1–O2 | 1.635 | 1.682 | O2–S1–O5 | 102.857 | 94.369 | O5–S1–O2–N9 | 179.976 | -176.002 |
| S1–O5 | 1.613 | 1.611 | O2–S1–O6 | 108.410 | 109.111 | O6–S1–O2–N9 | 66.217 | 74.500 |
| S1–O6 | 1.446 | 1.439 | O2–S1–O7 | 109.345 | 109.406 | O7–S1–O2–N9 | -68.668 | -62.237 |
| S1–O7 | 1.447 | 1.442 | O5–S1–O6 | 107.603 | 106.830 | S1–O2–N9–C11 | -122.032 | -111.954 |
| O2–N9 | 1.425 | 1.407 | O5–S1–O7 | 105.146 | 110.712 | S1–O2–N9–16 | 117.159 | 126.090 |
| O3–C16 | 1.221 | 1.197 | O6–S1–O7 | 121.813 | 122.697 | C13–N8–C12–C11 | 78.798 | 77.553 |
| O4–C17 | 1.228 | 1.217 | S1–O2–N9 | 113.296 | 110.089 | C16–N8–C12–C11 | -40.180 | -38.339 |
| N8–C12 | 1.464 | 1.492 | C12–N8–C13 | 113.886 | 109.092 | C12–N8–C13–C14 | -43.852 | -47.565 |
| N8–C13 | 1.486 | 1.493 | C12–N8–C16 | 104.837 | 104.982 | C12–N8–C13–C17 | 81.362 | 81.555 |
| N8–C16 | 1.453 | 1.435 | C13–N8–C16 | 108.936 | 108.305 | C16–N8–C13–C14 | 72.764 | 66.179 |
| N9–C11 | 1.459 | 1.499 | O2–N9–C11 | 114.408 | 116.136 | C16–N8–C13–C17 | -162.022 | -164.702 |
| N9–C16 | 1.429 | 1.421 | O2–N9–C16 | 110.426 | 112.257 | C12–N8–C16–O3 | -175.908 | -173.782 |
| N10–C17 | 1.369 | 1.356 | C11–N9–C16 | 107.030 | 105.838 | C12–N8–C16–N9 | 17.136 | 9.146 |
| C11–C12 | 1.519 | 1.525 | N9–C11–C12 | 98.689 | 94.907 | C13–N8–C16–O3 | 61.834 | 69.783 |
| C11–C15 | 1.525 | 1.518 | N9–C11–C15 | 110.432 | 112.383 | C13–N8–C16–N9 | -105.122 | -107.288 |
| C13–C14 | 1.553 | 1.534 | C12–C11–C15 | 106.401 | 108.452 | O2–N9–C11–C12 | -161.009 | -171.851 |
| C13–C17 | 1.557 | 1.551 | N8–C12–C11 | 99.842 | 99.922 | O2–N9–C11–C15 | -49.802 | -59.585 |
| C14–C15 | 1.359 | 1.342 | N8–C13–C14 | 107.656 | 111.703 | C16–N9–C11–C12 | -38.338 | -46.559 |
| C14–C18 | 1.502 | 1.502 | N8–C13–C17 | 112.336 | 109.994 | C16–N9–C11–C15 | 72.869 | 65.707 |
| **--** | **--** | **--** | C14–C13–C17 | 113.140 | 115.116 | O2–N9–C16–O3 | -27.231 | -24.768 |
| **--** | **--** | **--** | C13–C14–C15 | 121.429 | 119.475 | O2–N9–C16–N8 | 139.465 | 152.316 |
| **--** | **--** | **--** | C13–C14–C18 | 117.014 | 117.627 | C11–N9–16–O3 | -152.347 | -152.419 |
| **--** | **--** | **--** | C15–C14–C18 | 121.382 | 122.731 | C11–N9–16–N8 | 14.348 | 24.665 |
| **--** | **--** | **--** | C11–C15–C14 | 120.774 | 121.522 | N9–C11–C12–N8 | 47.241 | 49.877 |
| **--** | **--** | **--** | O3–C16–N8 | 125.526 | 127.054 | C15–C11–C12–N8 | -67.159 | -65.689 |
| **--** | **--** | **--** | O3–C16–N9 | 127.042 | 126.729 | N9–C11–C15–C14 | -74.021 | -74.842 |
| **--** | **--** | **--** | N8–C16–N9 | 105.968 | 106.146 | C12–C11–C15–C14 | 32.101 | 28.746 |
| **--** | **--** | **--** | O4–C17–N10 | 119.645 | 123.718 | N8–C13–C14–C15 | 1.296 | 5.587 |
| **--** | **--** | **--** | O4–C17–C13 | 124.043 | 122.487 | N8–C13–C14–C18 | -173.967 | -169.844 |
| **--** | **--** | **--** | N10–C17–C13 | 116.301 | 113.743 | C17–C13–C14–C15 | -123.436 | -120.783 |
| **--** | **--** | **--** | **--** | **--** | **--** | C17–C13–C14–C18 | 61.301 | 63.786 |
| **--** | **--** | **--** | **--** | **--** | **--** | N8–C13–C17–O4 | -177.731 | -153.323 |
| **--** | **--** | **--** | **--** | **--** | **--** | N8–C13–C17–N10 | 3.513 | 29.188 |
| **--** | **--** | **--** | **--** | **--** | **--** | C14–C13–C17–O4 | -55.578 | -26.083 |
| **--** | **--** | **--** | **--** | **--** | **--** | C14–C13–C17–N10 | 125.666 | 156.429 |
| **--** | **--** | **--** | **--** | **--** | **--** | C13–C14–C15–C11 | 2.528 | 3.089 |
| **--** | **--** | **--** | **--** | **--** | **--** | C18–C14–C15–C11 | 177.584 | 178.276 |
| **A7** | | | | | | | | |
| S1–O2 | 1.635 | 1.682 | O2–S1–O5 | 102.857 | 94.369 | O5–S1–O2–N9 | 179.976 | -176.002 |
| S1–O5 | 1.613 | 1.611 | O2–S1–O6 | 108.410 | 109.111 | O6–S1–O2–N9 | 66.217 | 74.500 |
| S1–O6 | 1.446 | 1.439 | O2–S1–O7 | 109.345 | 109.406 | O7–S1–O2–N9 | -68.668 | -62.237 |
| S1–O7 | 1.447 | 1.442 | O5–S1–O6 | 107.603 | 106.830 | S1–O2–N9–C11 | -122.032 | -111.954 |
| O2–N9 | 1.425 | 1.407 | O5–S1–O7 | 105.146 | 110.712 | S1–O2–N9–16 | 117.159 | 126.090 |
| O3–C16 | 1.221 | 1.197 | O6–S1–O7 | 121.813 | 122.697 | C13–N8–C12–C11 | 78.798 | 77.553 |
| O4–C17 | 1.228 | 1.217 | S1–O2–N9 | 113.296 | 110.089 | C16–N8–C12–C11 | -40.180 | -38.339 |
| N8–C12 | 1.464 | 1.492 | C12–N8–C13 | 113.886 | 109.092 | C12–N8–C13–C14 | -43.852 | -47.565 |
| N8–C13 | 1.486 | 1.493 | C12–N8–C16 | 104.837 | 104.982 | C12–N8–C13–C17 | 81.362 | 81.555 |
| N8–C16 | 1.453 | 1.435 | C13–N8–C16 | 108.936 | 108.305 | C16–N8–C13–C14 | 72.764 | 66.179 |
| N9–C11 | 1.459 | 1.499 | O2–N9–C11 | 114.408 | 116.136 | C16–N8–C13–C17 | -162.022 | -164.702 |
| N9–C16 | 1.429 | 1.421 | O2–N9–C16 | 110.426 | 112.257 | C12–N8–C16–O3 | -175.908 | -173.782 |
| N10–C17 | 1.369 | 1.356 | C11–N9–C16 | 107.030 | 105.838 | C12–N8–C16–N9 | 17.136 | 9.146 |
| C11–C12 | 1.519 | 1.525 | N9–C11–C12 | 98.689 | 94.907 | C13–N8–C16–O3 | 61.834 | 69.783 |
| C11–C15 | 1.525 | 1.518 | N9–C11–C15 | 110.432 | 112.383 | C13–N8–C16–N9 | -105.122 | -107.288 |
| C13–C14 | 1.553 | 1.534 | C12–C11–C15 | 106.401 | 108.452 | O2–N9–C11–C12 | -161.009 | -171.851 |
| C13–C17 | 1.557 | 1.551 | N8–C12–C11 | 99.842 | 99.922 | O2–N9–C11–C15 | -49.802 | -59.585 |
| C14–C15 | 1.359 | 1.342 | N8–C13–C14 | 107.656 | 111.703 | C16–N9–C11–C12 | -38.338 | -46.559 |
| C14–C18 | 1.502 | 1.502 | N8–C13–C17 | 112.336 | 109.994 | C16–N9–C11–C15 | 72.869 | 65.707 |
| **--** | **--** | **--** | C14–C13–C17 | 113.140 | 115.116 | O2–N9–C16–O3 | -27.231 | -24.768 |
| **--** | **--** | **--** | C13–C14–C15 | 121.429 | 119.475 | O2–N9–C16–N8 | 139.465 | 152.316 |
| **--** | **--** | **--** | C13–C14–C18 | 117.014 | 117.627 | C11–N9–16–O3 | -152.347 | -152.419 |
| **--** | **--** | **--** | C15–C14–C18 | 121.382 | 122.731 | C11–N9–16–N8 | 14.348 | 24.665 |
| **--** | **--** | **--** | C11–C15–C14 | 120.774 | 121.522 | N9–C11–C12–N8 | 47.241 | 49.877 |
| **--** | **--** | **--** | O3–C16–N8 | 125.526 | 127.054 | C15–C11–C12–N8 | -67.159 | -65.689 |
| **--** | **--** | **--** | O3–C16–N9 | 127.042 | 126.729 | N9–C11–C15–C14 | -74.021 | -74.842 |
| **--** | **--** | **--** | N8–C16–N9 | 105.968 | 106.146 | C12–C11–C15–C14 | 32.101 | 28.746 |
| **--** | **--** | **--** | O4–C17–N10 | 119.645 | 123.718 | N8–C13–C14–C15 | 1.296 | 5.587 |
| **--** | **--** | **--** | O4–C17–C13 | 124.043 | 122.487 | N8–C13–C14–C18 | -173.967 | -169.844 |
| **--** | **--** | **--** | N10–C17–C13 | 116.301 | 113.743 | C17–C13–C14–C15 | -123.436 | -120.783 |
| **--** | **--** | **--** | **--** | **--** | **--** | C17–C13–C14–C18 | 61.301 | 63.786 |
| **--** | **--** | **--** | **--** | **--** | **--** | N8–C13–C17–O4 | -177.731 | -153.323 |
| **--** | **--** | **--** | **--** | **--** | **--** | N8–C13–C17–N10 | 3.513 | 29.188 |
| **--** | **--** | **--** | **--** | **--** | **--** | C14–C13–C17–O4 | -55.578 | -26.083 |
| **--** | **--** | **--** | **--** | **--** | **--** | C14–C13–C17–N10 | 125.666 | 156.429 |
| **--** | **--** | **--** | **--** | **--** | **--** | C13–C14–C15–C11 | 2.528 | 3.089 |
| **--** | **--** | **--** | **--** | **--** | **--** | C18–C14–C15–C11 | 177.584 | 178.276 |
